# Human Cystathionine γ-Lyase Is Inhibited by *s*-Nitrosation: A New Crosstalk Mechanism between NO and H_2_S

**DOI:** 10.3390/antiox10091391

**Published:** 2021-08-30

**Authors:** Dalila G. F. Fernandes, João Nunes, Catarina S. Tomé, Karim Zuhra, João M. F. Costa, Alexandra M. M. Antunes, Alessandro Giuffrè, João B. Vicente

**Affiliations:** 1 Instituto de Tecnologia Química e Biológica António Xavier (ITQB NOVA), 2780-157 Oeiras, Portugal; dalilagfhf@itqb.unl.pt (D.G.F.F.); cstome@farm-id.pt (C.S.T.); jmcosta@itqb.unl.pt (J.M.F.C.); 2 Centro de Química Estrutural, Instituto Superior Técnico, ULisboa, 1049-001 Lisboa, Portugal; joaopcnunes@tecnico.ulisboa.pt (J.N.); alexandra.antunes@tecnico.ulisboa.pt (A.M.M.A.); 3 Instituto de Biologia Experimental e Tecnológica, 2780-157 Oeiras, Portugal; 4 CNR Institute of Molecular Biology and Pathology, I-00185 Rome, Italy; karim.zuhra@unifr.ch; 5 Department of Biochemical Sciences, Sapienza University of Rome, I-00185 Rome, Italy

**Keywords:** hydrogen sulfide, *s*-nitrosoglutathione, gasotransmitters, cystathionine γ-lyase, signaling, crosstalk

## Abstract

The ‘gasotransmitters’ hydrogen sulfide (H_2_S), nitric oxide (NO), and carbon monoxide (CO) act as second messengers in human physiology, mediating signal transduction via interaction with or chemical modification of protein targets, thereby regulating processes such as neurotransmission, blood flow, immunomodulation, or energy metabolism. Due to their broad reactivity and potential toxicity, the biosynthesis and breakdown of H_2_S, NO, and CO are tightly regulated. Growing evidence highlights the active role of gasotransmitters in their mutual cross-regulation. In human physiology, the transsulfuration enzymes cystathionine β-synthase (CBS) and cystathionine γ-lyase (CSE) are prominent H_2_S enzymatic sources. While CBS is known to be inhibited by NO and CO, little is known about CSE regulation by gasotransmitters. Herein, we investigated the effect of *s*-nitrosation on CSE catalytic activity. H_2_S production by recombinant human CSE was found to be inhibited by the physiological nitrosating agent *s*-nitrosoglutathione (GSNO), while reduced glutathione had no effect. GSNO-induced inhibition was partially reverted by ascorbate and accompanied by the disappearance of one solvent accessible protein thiol. By combining differential derivatization procedures and mass spectrometry-based analysis with functional assays, seven out of the ten protein cysteine residues, namely Cys84, Cys109, Cys137, Cys172, Cys229, Cys307, and Cys310, were identified as targets of *s*-nitrosation. By generating conservative Cys-to-Ser variants of the identified *s*-nitrosated cysteines, Cys137 was identified as most significantly contributing to the GSNO-mediated CSE inhibition. These results highlight a new mechanism of crosstalk between gasotransmitters.

## 1. Introduction

Hydrogen sulfide (H_2_S), nitric oxide (NO), and carbon monoxide (CO), the three gasotransmitters in mammalian physiology are gaseous molecules endogenously produced to act as second messengers and mediate signal transduction [1,2,3]. This signaling function is achieved by targeting proteins, namely via binding to metal centers and post-translational modification of specific residues (reviewed e.g., in [4,5]). The ability of gasotransmitters to interact with proteins underlies both their physiological roles and potential toxicity. Indeed, depending on their concentration, beneficial or deleterious effects for human health may be elicited.

Within its physiological concentration range, H_2_S is known to regulate several physiological processes such as neurotransmission, inflammation, immunomodulation, blood flow, apoptosis, and energy metabolism, whereas dysregulation of H_2_S homeostasis was found to be implicated in several diseases including cancer and cardiovascular and neurological disorders [5,6]. This dual role of H_2_S requires a tight balance of its homeostasis, which is maintained by several finely regulated enzymes responsible for its synthesis and breakdown. The transsulfuration enzymes cystathionine β-synthase (CBS) and cystathionine γ-lyase (CSE), and 3-mercaptopyruvate sulfurtransferase (MST) synthesize H_2_S, while H_2_S degradation is mainly catalyzed by the mitochondrial sulfide oxidation pathway.

CSE is a homotetrameric enzyme of 44 kDa subunits. It contains an N-terminal catalytic domain that binds the cofactor pyridoxal-5′-phosphate (PLP) through covalent linkage with a lysine (K212) forming a Schiff base. CSE contains ten cysteine residues including two CXXC motifs. Despite the proximity between several Cys residues within the CSE structure, no disulfide bonds are formed, which constitutes an unusually high number of virtually exposed cysteine thiols. CSE catalyzes the second and final step of the methionine cycle reverse transsulfuration branch. In the first step, CBS condenses homocysteine and serine into cystathionine. Then, CSE catalyzes the conversion of cystathionine to cysteine, α-ketobutyrate, and ammonia. CSE thus contributes to homocysteine homeostasis and provides a cysteine source under high methionine dietary intake. Due to its substrate promiscuity, CSE is also able to use different combinations of sulfur-containing substrates to generate H_2_S [5,7]. Indeed, it is the most efficient H_2_S-generating enzyme at physiologic (homo)cysteine levels [8,9]. Mutations identified in patients with the rare inherited error of metabolism cystathioninuria lead to CSE variants with amino acid substitutions (p.T67I and p.Q240E) that result in lower PLP cofactor affinity and impaired enzymatic activity. Both mutations affect structural elements located in the vicinity of the PLP active site [10]. Despite being a rare disease (estimated prevalence of 1:14,000 live births [11]), cystathioninuria is a co-morbidity of several other diseases such as diabetes insipidus, Down’s syndrome, neuroblastoma, hepatoblastoma, and celiac disease [11,12].

Data on CSE regulation are scarce and point mainly to mechanisms operating at the transcriptional level [13]. Studies revealed upregulation of CSE expression by TNFα-mediated recruitment of the SP1 transcription factor [14], LPS-mediated recruitment of NF-κB [15,16], endoplasmatic reticulum (ER) stress-mediated recruitment of ATF4 transcription factor [17], and a tissue-specific up/downregulation in diabetes models [18,19], suggesting modulation in response to stimuli such as inflammation, apoptosis, and oxidative stress. Modulation of CSE transcription by hypoxia [20] and exogenous H_2_S [21] has also been reported. Regulatory mechanisms at the protein level are not as widely explored yet. In vitro SUMOylation of CSE has been reported [22], although the functional implications remain to be determined. Recently, CSE has been shown to be impaired by nitration of tyrosine residues promoted by excess homocysteine as a result of high dietary methionine in mice [23]. Notably, in the same report, Luo et al. demonstrated that seven cysteine residues (Cys84, Cys109, Cys172, Cys229, Cys252, Cys307, and Cys310) in human CSE expressed in HEK293 cells can be targeted by persulfidation. Furthermore, except for Cys172 and Cys310, this cysteine modification appears to be important for the CSE H_2_S-synthesizing activity [23].

The biological roles of H_2_S are interconnected with those of NO and CO through a mechanistic crosstalk (reviewed e.g., in [5,24]). In some cases, the gasotransmitters can undergo direct chemical reactions between themselves, producing reactive species that further propagate signal transduction [5,25,26,27]. In other cases, they independently act on the same pathways, having concordant or opposing effects. More complex is the interplay that the three gasotransmitters establish through modulation of their own metabolic pathways. Examples of the latter case are the reversible inhibition of H_2_S production by NO and CO binding to the CBS heme iron [9,28,29,30,31,32,33,34], and the inhibition of the mitochondrial sulfide oxidizing pathway suggested to occur via NO- and CO-mediated inhibition of cytochrome *c* oxidase (CcOX), leading to quinol oxidation impairment uphill of the electron transport chain [5,35]. As for CSE, while various studies have reported on the relevance of CSE-derived H_2_S for the control of eNOS in different (patho)physiological contexts (e.g., [36,37,38]), little is known about how NO and related reactive nitrogen species modulate CSE function. Asimakopoulou and co-workers reported on CSE inhibition by the NO donor diethylamine NONOate and hypothesized the possibility of cysteine *s*-nitrosation [39]. Moreover, as described above, CSE nitration modulated by excess homocysteine negatively impacts CSE activity [23].

*S*-nitrosation is a prominent post-translational modification very relevant in health and disease (reviewed e.g., in [40]). It can occur via reaction of NO with metal-oxidized cysteinyl radicals, or via transfer of a nitrosonium ion (NO^+^) from low molecular thiols such as GSNO (transnitrosation) [41,42]. *S*-nitrosation/denitrosation of protein thiols leads to changes in enzymatic activity, protein conformation, subcellular localization, and protein–protein interactions [43]. Here, by investigating the effect of *s*-nitrosation on human CSE function, we disclose a new crosstalk mechanism between H_2_S and NO.

## 2. Materials and Methods

### 2.1. Materials

Tris hydrochloride (#9090.3), di-potassium hydrogen phosphate (#6875.3), 1,4-dithiothreitol (DTT, #6908.3), tris(2-carboxyethyl)phosphine (TCEP, #HN95.2), and ethylenediaminetetraacetic acid (EDTA, #8040.2) were purchased from Carl Roth GmbH + Co. KG (Karlsruhe, Germany). Potassium phosphate monobasic (#60229) was purchased from Fluka Analytical (Sigma-Aldrich, Steinheim, Germany). Glycerol (#24388.295) was purchased from VWR International (Leuven, Belgium). Sodium chloride (#1.06404.5000) and imidazole (#1.04716.1000) were purchased from Merck KGaA (Darmstadt, Germany). The protease inhibitor cocktail cOmplete Tablets Mini EDTA-free EASYpack (#04693159001) was purchased from Roche (Mannheim, Germany). Lysozyme (#62971), deoxyribonuclease I (DNAse I, #DN25), pyridoxal 5′-phosphate hydrate (PLP, #P9255), l-cysteine hydrochloride (#C1276), l-homocysteine thiolactone hydrochloride (#H6503), O-(carboxymethyl)hydroxylamine hemihydrochloride (AOAA, #C13408), (+)-sodium l-ascorbate (#11140), reduced l-glutathione (#G4251), 7-azido-4-methylcoumarin (AzMc, #802409), 5,5′-dithiobis-(2-nitrobenzoic acid) (DTNB, #D8130), iodoacetamide (#I1149), acrylamide (#8.00830), Trizma^®®^ base (#T1503), and urea (#GE17-1319-01) were purchased from Sigma-Aldrich (St. Louis, MO, USA). *S*-nitrosoglutathione (GSNO) was purchased from Sigma-Aldrich (#N4148) and Santa Cruz Biotechnology (#sc-200349C). Trypsin (#V5280) was purchased from Promega (Madisson, WI, USA) and formic acid (OptimaTM #A117-50) from Thermo Fisher Scientific (Waltham, MA, USA).

### 2.2. Protein Expression and Purification

The gene encoding wild-type (WT) CSE was synthesized (Genscript, The Netherlands) including an N-terminal His6 tag followed by two recognition sites for the TEV and the 3C protease, respectively. The synthetic gene was cloned between the NcoI and BamHI sites of pET-28b. The C70S, C84S, C109S, C137S, C172S, C229S, C307S, and C310S variants were generated by site-directed mutagenesis using the WT-expressing vector as a template (Genscript, The Netherlands). All cloned sequences can be found in the Supplementary Materials.

Recombinant human CSE was expressed and purified essentially as in [44] with a few modifications. Cell pellets were resuspended in buffer A (50 mM Tris-HCl buffer, pH 7.5, 300 mM NaCl, 10% glycerol, 500 μM TCEP), supplemented with 1 mg/mL lysozyme, DNAse I, and cOmplete EDTA-free Protease Inhibitor Cocktail Tablet (Roche), and lysed by five cycles of sonication (each cycle: 30 s, 0.6 amplitude and 50% duty cycle). After centrifugation at 26,500 g, 20 min, 4 °C, the soluble lysate was injected in a HisTrap™ FF Crude column (GE Healthcare, Carnaxide, Portugal) equilibrated with buffer A containing 20 μM PLP and 10 mM imidazole, and elution was performed with a 10–500 mM imidazole gradient (in the same buffer). The fractions containing CSE were further purified by size exclusion chromatography in a HiLoad™ 16/600 Superdex™ 200 column (GE) equilibrated in buffer B (20 mM Tris-HCl, pH 7.5, 150 mM NaCl, 10% glycerol, 100 μM TCEP, 20 μM PLP). WT CSE and all protein variants eluted essentially in their tetrameric form. CSE was concentrated by ultra-filtration with Amicon-30kDa Ultra-15 centrifugal filter units (Merck, Darmstadt, Germany) and protein batches were flash frozen in liquid nitrogen and stored at −80 °C.

### 2.3. Differential Scanning Fluorimetry

The effect of Cys-to-Ser substitutions on protein stability was assessed by dye-free differential scanning fluorimetry in a NanoTemper Prometheus NT.48 (NanoTemper, Munich, Germany), monitoring the intrinsic tryptophan fluorescence as a function of linearly increasing temperature. Each CSE variant was tested (in triplicates) at 0.5 mg·mL^−1^ in buffer B. A 2-min hold step at 20 °C was followed by a 1 °C·min^−1^ linear temperature gradient, measuring in simultaneous fluorescence emission at 330 nm and 350 nm, with fluorescence excitation at 275 nm. Data are represented as the ratio between the fluorescence emission at 330 nm and that at 350 nm, as a function of temperature. The melting temperature (*T*_m_) was determined from the first derivative of the thermal denaturation curves.

### 2.4. Spectrophotometric Measurements

UV–Visible absorption spectra were recorded in a Shimadzu UV-1800 spectrophotometer (Shimadzu Corporation, Kyoto, Japan) coupled to a Shimadzu TCC-100 Peltier temperature controller (Shimadzu Corporation, Kyoto, Japan) and a Starna ‘Spinette’ electronic cell stirrer (Starna Analytical Accessories, Starna Analytical Accessories, Essex, UK) using Hellma^®®^ Analytics SUPRASIL 1-cm path length quartz cuvettes.

### 2.5. Enzymatic Activity Assays

CSE activity assays were carried out as described in [45,46] using the H_2_S-selective fluorogenic probe 7-azido-4-methylcoumarin (AzMC). Prior to activity measurements, CSE was reacted with GSNO and/or ascorbate. The reaction mixtures (250 μL per reaction) containing 20 μg CSE and 50 μM PLP in 200 mM Tris-HCl buffer, pH 8.0, 0.1 mM EDTA, were prepared in micro-tubes in triplicate. EDTA was added to the reaction buffer to prevent cleavage of GSNO by reaction with contaminant transition metals, with concomitant release of NO [47,48,49]. In this manner, the possible reaction of NO with CSE-generated H_2_S is also prevented, thereby excluding the formation to any significant extent of hybrid H_2_S/NO species that could themselves react with CSE [26]. Under the tested conditions, given the reactants’ concentrations and ratios and the timescale of the experiments, the possible reaction of H_2_S with GSNO is also unlikely to interfere with the measurements [50,51,52]. GSNO 0.5 mM (or buffer in control samples) was added to the reaction mixture and incubated for 30 min. The concentration of GSNO stocks was checked spectrophotometrically prior to each experiment. Ascorbate 2 mM (or buffer) was then added to the reaction mixture and incubated for 30 min. Alternatively, as a control, GSNO and ascorbate were previously reacted for 30 min, and afterward incubated with the reaction mixture for 30 min. All procedures were performed at room temperature and protected from light. Each reaction mix was transferred to a 96-well black plate (Corning Costar^®®^) and 10 or 50 μM AzMC was added to each well. The plate was incubated at 37 °C for 10 min. CSE activity was triggered by the addition of l-homocysteine and l-cysteine (both at 2.5 mM final concentration). Fluorescence was monitored in a Thermo Scientific Appliskan^®®^ microplate reader at 37 °C for 1.5 h (λ_excitation_ = 340 nm; λ_emission_ = 460 nm) or a TECAN Spark 10M. A sample containing 1 mM aminooxyacetic acid (AOAA) was used as a positive inhibition control. Enzyme activity was calculated from the slope of the absorbance increase, after blank subtraction. The time interval of the curve from which the slope was extracted corresponded to the first derivative maximum of the curve collected with unreacted CSE. All samples (GSNO-, ascorbate-, and AOAA-reacted) were analyzed in such time intervals, and relative activities were calculated using unreacted CSE as the reference.

### 2.6. Determination of the Number of Free Exposed Thiols in Human Cystathionine γ-Lyase

The number of free exposed cysteine thiols in CSE was determined using DTNB. DTNB stocks were prepared at 10 mM in 100 mM phosphate buffer, pH 8.0, containing 1 mM EDTA. Prior to thiol quantitation, CSE was reacted with GSNO and/or ascorbate. Reaction mixtures were prepared in 200 mM Tris-HCl buffer, pH 8.0, to a final protein concentration of 8 μM and a final volume of 500 μL. A first incubation was performed with 0.5 mM GSNO (or buffer in control samples) for 30 min, followed by buffer exchange with a PD MiniTrap™ G-25 desalting column (GE Healthcare) equilibrated in 200 mM Tris pH 8.0. Only the initial 500 μL of eluate were recovered to avoid residual GSNO present at the end of the elution peak. A second incubation was then performed with 2 mM ascorbate (or buffer) for 30 min, followed by buffer exchange with a PD MiniTrap™ G-25 column equilibrated with 100 mM phosphate buffer pH 8.0, 1 mM EDTA. Only the initial 500 μL of the eluate was again recovered to avoid contamination by residual ascorbate. Alternatively, GSNO and ascorbate were previously reacted for 30 min, then incubated with the reaction mix for 30 min, and finally desalted in 100 mM phosphate buffer pH 8.0, 1 mM EDTA. All incubations and desalting steps were performed at room temperature, protected from light. Incubations were also undertaken with 300 μL of mineral oil above the aqueous medium to limit air exposure and ascorbate oxidation. A quantity of 10 μL of a 10 mM DTNB stock was added to 450 μL of eluate and incubated for 15 min at 20 °C. Spectra were recorded between 250 and 600 nm. Spectra were also recorded for the protein samples (before DTNB addition) and for DTNB alone (in 100 mM phosphate buffer pH 8.0, 1 mM EDTA). The number of free thiols was calculated employing Equation (1):

(1) $$\mathrm{SH}=\;\frac{{\mathrm{Abs}}_{412\mathrm{nm}}^{\mathrm{protein}+\mathrm{DTNB}}-{\;\mathrm{Abs}}_{412\mathrm{nm}}^{\mathrm{protein}}-{\;\mathrm{Abs}}_{412\mathrm{nm}}^{\mathrm{DTNB}}}{14,150\;\times \left[\mathrm{CSE}\right]}$$

where Abs_412nm_ corresponds to absorbance values at 412 nm, 14,150 M^−1^·cm^−1^ is the molar extinction coefficient of TNB, and [CSE] is the protein concentration in the cuvette (expressed in M).

### 2.7. Effect of CSE S-Nitrosation/Denitrosation on PLP Cofactor Load

The effect of GSNO-mediated WT CSE nitrosation/denitrosation on the PLP moiety load was evaluated by recording UV–Visible absorption spectra. CSE was diluted to 6 μM in 200 mM Tris-HCl buffer at pH 8.0, incubated at 20 °C with 40 μM GSNO for 30 min, and then with 2 mM ascorbate for another 30 min. Spectra were recorded at different time points prior to and after addition of ascorbate. Control spectra were obtained for CSE incubated with reduced glutathione and/or sodium nitrite.

### 2.8. Liquid Chromatography-High Resolution Mass Spectrometry (LC-HRMS) Analysis

Mass spectrometry (MS)-based analysis was used to identify modified cysteine residues. Prior to MS analysis, CSE (WT and Cys-to-Ser variants) was reacted with GSNO and/or ascorbate essentially as described above. The reaction mixtures, containing 8 μM CSE, were prepared in 200 mM Tris-HCl buffer, pH 8.0. Two versions of a dual-derivatization procedure (Supplementary Figures S1 and S2) were designed and employed to distinguish *s*-nitrosated cysteines from other cysteine forms (particularly reduced thiols and disulfide bonds). Unless stated otherwise, all incubations, buffer exchange, and concentration steps were performed at room temperature, and samples were protected from light. In the first strategy (Supplementary Figures S1 and S2), exposed non-nitrosated cysteine thiols were covalently modified by incubation with 55 mM iodoacetamide (IAA) for 45 min. IAA excess was removed through a buffer exchange step using a PD MiniTrap™ G-25 column (GE) equilibrated and eluted with 200 mM Tris-HCl buffer, pH 8.0. Afterward, the protein sample was concentrated using a Microcon-30kDa centrifugal filter unit (Merck). The protein sample was then incubated for 30 min with ascorbate (5 mM) to reduce possible *s*-nitrosated cysteines; excess ascorbate was removed by buffer exchange and concentration as described above. Subsequently, ascorbate-reduced cysteines were covalently modified with acrylamide (AA, 55 mM, incubation for 45 min). AA excess was removed by buffer exchange and concentration as before. The protein was then denatured and its putative disulfide bridges reduced, respectively, by incubation with 8 M urea and 1 mM DTT for 30 min at 37 °C. Free/exposed cysteines in unfolded CSE were labelled with 55 mM IAA for 45 min. Excess urea, DTT, and IAA were removed by buffer exchange and concentration as described above. In the second version of this derivatization procedure (Supplementary Figures S1 and S2), we included 6 M urea from the first derivatization step with IAA to enable simultaneous and complete labelling of buried cysteines besides the exposed ones. In fact, 6 M urea was always present in derivatization steps from that point on, namely: reduction with ascorbate, derivatization with AA, and all PD10 buffer exchange and concentration steps in between. Urea was only removed in the final AA removal step, which was done with a PD MiniTrap™ G-25 equilibrated and eluted with 50 mM ammonium bicarbonate buffer, pH 8.5. Protein samples were quantitated with the Bradford assay. Protein digestion was achieved by adding trypsin at a trypsin:CSE ratio of 15:1 (mass/mass), incubating overnight at 37 °C under stirring, and stopping the reaction by the addition of 10% formic acid. Following protein digestion, the peptides were analyzed by liquid chromatography (Ultra High Performance Liquid Chromatography system, Bruker Elute, Mannheim, Germany) interfaced with a Bruker Impact II quadrupole time-of-flight mass spectrometer equipped with an electrospray source (Bruker Daltoniks, Mannheim, Germany). Chromatographic separation was performed on an Acclaim PepMap C18 column (1.0 mm × 150 mm, 3 μm particle size; Thermo Scientific, Oeiras, Portugal). The mobile phase consisted of water containing 0.1% formic acid (A) and acetonitrile containing 0.1% formic acid (B). The elution conditions were as follows: 0.2% B for 1 min, 0.2–46.2% B over 59 min, 46.2–90% B over 1 min, 90% B for 4 min, 90–0.2% B over 1 min, and 0.2% B for 14 min. The injection volume was 10 μL, the flow rate was 100 μL·min^−1^, and the column was maintained at 40 °C. Quality control samples (a tryptic peptide digest of bovine serum albumin) were analyzed along with the analytical runs (after every 10 samples) in order to check the consistency of analysis regarding signal intensity and retention time deviations. An ESI-L Low Concentration Tuning Mix (Agilent Technologies, Santa Clara, CA, USA) was used during the analysis for spectrum calibration. Sample analysis was performed by data-dependent acquisition (auto MSMS mode) in the 300–2200 m/z range with a 2 Hz rate and by a dynamic method with a fixed cycle time of 3 s. The MS source parameters were set as follows: dry gas heater temperature, 200 °C; dry gas flow, 8 L·min^−1^; and capillary voltage, 4500 V.

### 2.9. Database Searching and Processing

The acquired MS data files of the samples were converted to *.mgf format using the Compass DataAnalysis software (Bruker Daltonics). Two distinct search engines were used for peptide identification: Mascot (v2.6, Matrix Science Ltd., London, UK) [53] and MaxQuant [54]. Search parameters were the same for the two methods and included precursor ion mass tolerance = 10 ppm, fragment ion mass tolerance = 20 ppm, number of missed cleavages ≤2, and variable amino acid modifications: oxidation of methionine, carbamidomethylation of cysteines, and acrylamide incorporation (mass increment of 71.0371 Da) into cysteines. The acquired MS/MS spectra were searched against a database containing only the human CSE protein sequence, obtained from Uniprot [55]. All spectra corresponding to acrylamide-modified peptides were manually checked. The resulting database searches containing MS/MS spectra were then used to generate spectral libraries in the Skyline software [56] prior to MS1 filtering analyses.

### 2.10. Statistical Analysis

At least three independent experiments were carried out for each type of assay. Data are presented as mean ± standard error (SE). For comparison of groups of more than two datasets, a one-way analysis of variance (ANOVA) with Dunnett’s multiple comparisons test was performed using GraphPad Prism 8.3.1. software. We also used unpaired Student’s *t* test to analyze data with only two sets, particularly when analyzing the number of free exposed thiols in each CSE variant by comparing the untreated with the GSNO-treated protein.

## 3. Results

### 3.1. Effect of GSNO on CSE Enzymatic Activity and Cofactor Load

**Figure 1:**
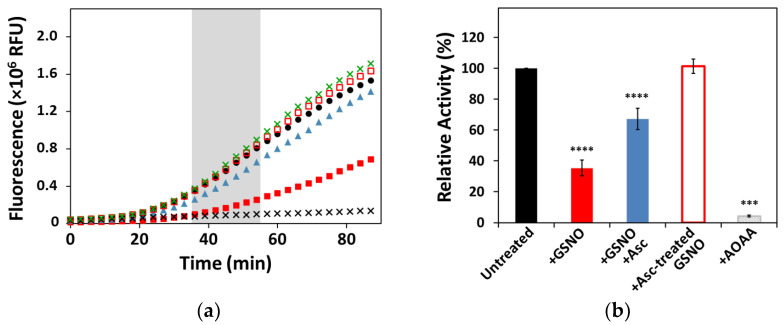
Effect of GSNO on CSE enzymatic activity. H_2_S production by CSE was measured at 37 °C, monitoring AzMc fluorescence in a plate reader. Reaction mixtures contained CSE (80. μg·mL^−1^) in 200 mM Tris-HCl pH 8.0, 50 μM PLP, 2.5 mM l-cysteine, and 2.5 mM l-homocysteine. CSE activity was analyzed for the as isolated protein (black circles; Ctrl) and for CSE incubated with: GSNO (red full squares; +GSNO); GSNO and subsequently ascorbate (blue triangles; +GSNO +Asc); GSNO preincubated with ascorbate (red hollow squares; +Asc-treated GSNO); ascorbate alone (green×); and AOAA (black×). (**a**) Representative reaction traces, highlighting the region of the curve where CSE activity was measured from the calculated slope (grey bar). (**b**) Histogram representing relative activities normalized to unreacted CSE. Error bars represent the standard error (SE), where *n* ≥ 3. Differences between groups were assessed by one-way ANOVA, **** *p* < 0.0001, *** *p* < 0.001.

The H_2_S-generating activity of human CSE was measured prior to and after incubating the enzyme with *s*-nitrosoglutathione (GSNO) (Figure 1). The GSNO-reacted protein displayed 35 ± 5% activity compared to the unreacted enzyme. Control experiments were performed to evaluate the functional recovery of CSE upon reversal of *s*-nitrosation by excess ascorbate, which yielded 67 ± 7% of the control CSE activity. Pre-incubation of GSNO with ascorbate prior to the addition to CSE resulted in a fully active enzyme (101 ± 5%), similar to CSE incubated with reduced glutathione (GSH), which exhibited 103 ± 17% activity compared to unreacted CSE. Finally, CSE incubated with the inhibitor aminooxyacetic acid (AOAA) had close to null activity (4.3 ± 0.5%).

Since CSE activity depends on the PLP cofactor, the possible effect of GSNO incubation on the cofactor load was evaluated by collecting UV–Visible spectra of the enzyme prior to and after incubation with GSNO. In the as isolated enzyme, a single broad band with λ_max_ at ≈426 nm arising from PLP can be observed (Supplementary Figure S3), which is maintained upon prolonged incubation (>30 min) with GSNO alone, indicating no effect of GSNO on the PLP cofactor load. On addition of ascorbate to the GSNO-reacted CSE, the broad PLP band decreased in intensity by ≈20% compared to the unreacted enzyme.

### 3.2. Effect of GSNO on Free Exposed Cysteines in WT CSE

To determine the number of cysteine residues becoming nitrosated upon reaction with GSNO, we quantitated free exposed thiols in CSE prior to and after reaction with GSNO. Several measurements employing the DTNB assay on independent protein batches consistently revealed 2.4 ± 0.2 free cysteines in unreacted CSE. Upon incubation with GSNO, exposed thiols decreased to 1.6 ± 0.2 (*p* < 0.0001). Addition of ascorbate to revert or prevent *s*-nitrosation yielded 1.8 ± 0.1 (*p* < 0.0001) and 2.0 ± 0.1 (*p* < 0.001) free exposed cysteines, respectively.

### 3.3. Identification of Cys229 as the Exposed s-Nitrosated Cysteine

**Figure 2:**
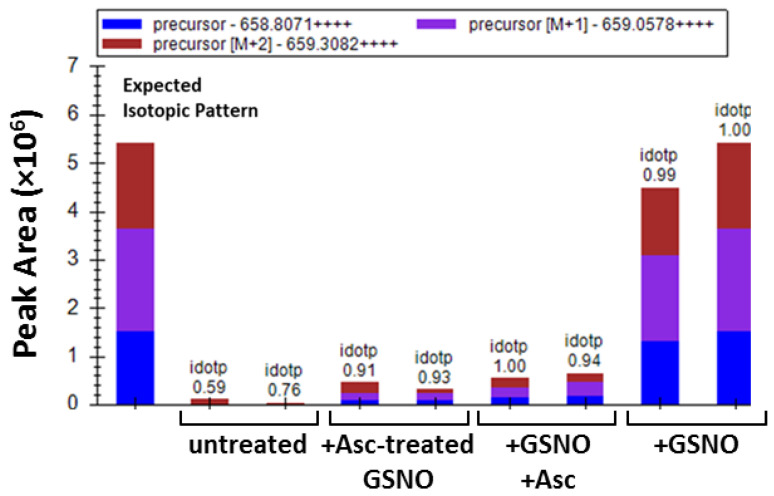
Identification of CSE exposed *s*-nitrosated cysteine(s) by mass spectrometry. Comparison of the peak areas corresponding to the tetra charged ion at *m/z* 658.81, which corresponds to the tryptic peptide ^213^YMNGHSDVVMGLVSVNCESLHNR^235^ with acrylamide (AA) incorporation at Cys229 in: GSNO-treated CSE (+GSNO); GSNO-treated CSE following incubation with ascorbate (+GSNO +Asc); GSNO pre-incubation with ascorbate before CSE treatment (+Asc-treated GSNO); untreated CSE (untreated). Two representative replicates of each sample are represented. The isotopic pattern ([M], [M + 1] and [M + 2] peaks) obtained is fully consistent with the one expected for the modified peptide. Idotp: dot product of the expected isotope distributions.

A mass spectrometry (MS)-based methodology was used to identify the sites of *s*-nitrosation, employing a dual derivatization strategy (reviewed e.g., in [57]), and using a sample pretreatment adapted from the FASILOX method [58]. The combined use of buffer exchange columns and small-scale ultra-filtration devices ensured the complete removal of the excess of labelling agents between steps, while avoiding dilution (Supplementary Figures S1 and S2). Using strategy 1, only Cys229 was identified as being modified with acrylamide (AA), thus indicating its *s*-nitrosation. Indeed, the tetra charged ion at m/z 658.8111 ± 6.1 ppm, corresponding to the tryptic peptide ^213^YMNGHSDVVMGLVSVNCESLHNR^235^ with an additional mass increment of 71.0371 Da, characteristic of AA incorporation, was consistently identified in all GSNO-treated samples (Figure 2). The *s*-nitrosated cysteine was confirmed to be Cys229 from the AA mass increment observed at y^7+^ (*m*/*z* 929.4265) ion, while ions y^6+^ (*m*/*z* 755.3816) and y^5+^ (*m*/*z* 626.3369) did not display this mass increment (Supplementary Figure S4). Importantly, although still detected in the samples where *s*-nitrosation was reverted or prevented by ascorbate, the peak areas of the AA-modified peptide were significantly higher in the replicates of GSNO-treated samples (Figure 2). Only trace amounts of AA-modified peptides were identified in the untreated CSE sample (Figure 2). Conversely, the above-mentioned tryptic peptide modified with IAA instead of AA was also identified in the GSNO-reacted samples, thereby evidencing partial *s*-nitrosation.

### 3.4. Cys229 s-Nitrosation Does Not Account for GSNO-Inhibition

**Figure 3:**
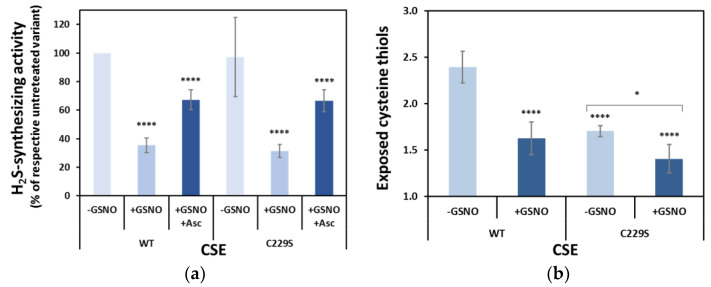
Effect of C229S substitution on GSNO-inhibition. (**a**) H_2_S production by GSNO-treated and GSNO/Asc-treated WT and C229S CSE, measured as described in the legend to Figure 1. Relative activities were normalized to the respective unreacted CSE variant, except for (**a**), where the activity of untreated C229S was normalized to that of untreated WT CSE. Ascorbate was added to the GSNO-modified CSE (+GSNO +Asc). Error bars represent the standard error (SE), where *n* ≥ 5. (**b**) number of free exposed cysteines in CSE as determined using DTNB for as isolated CSE (-GSNO) and upon incubation with GSNO (+GSNO). Error bars represent the standard error (SE), where *n* ≥ 4. One-way ANOVA was employed to compare differences between groups in both graphs, **** *p* < 0.0001. Student’s *t*-test was used to compare the exposed cysteine thiols of the C229S variant (**b**) with or without GSNO, * *p* < 0.05.

To further analyze the functional role of Cys229 as a site for *s*-nitrosation, we produced the recombinant C229S CSE variant. The stability of this variant appeared to be unaffected, as its melting temperature (*T*_m_) analyzed by dye-free differential scanning fluorimetry (nanoDSF) was 74.1 ± 0.1 °C compared to 73.2 ± 0.1 °C for WT CSE (Supplementary Figure S5). Under the same experimental conditions, WT and C229S CSE exhibited similar basal activity and similar GSNO-induced inhibitory effect and recovery by ascorbate (Figure 3a), therefore exempting Cys229 from having a major role in GSNO-inhibition of CSE. In addition, as expected, C229S revealed a lower number of free exposed cysteine thiols, i.e., 1.70 ± 0.06 (versus 2.4 ± 0.2 in the WT protein), which decreased to 1.41 ± 0.15 only after GSNO treatment (Figure 3b).

### 3.5. Identification of Buried s-Nitrosated Cysteines

**Figure 4:**
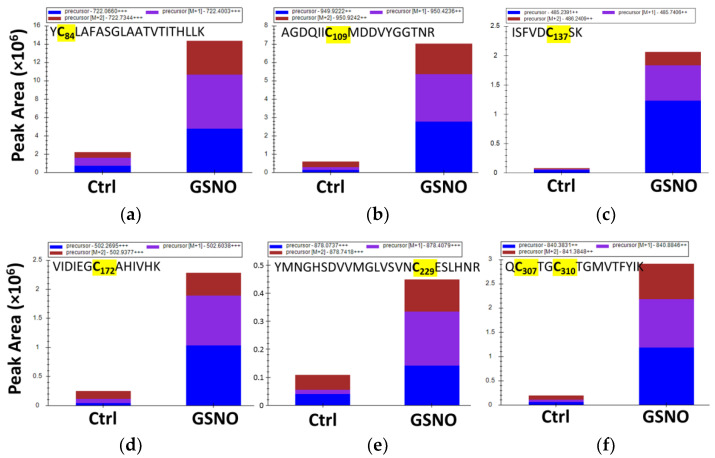
Identification of CSE buried *s*-nitrosated cysteine(s) by mass spectrometry. Comparison of peak areas from ions corresponding to the tryptic peptides containing Cys84 (**a**), Cys109 (**b**), Cys137 (**c**), Cys172 (**d**), Cys229 (**e**), and Cys307/Cys310 (**f**) with acrylamide (AA) incorporation, in GSNO-treated and Ctrl samples. The isotopic pattern ([M], [M + 1] and [M + 2] peaks) obtained is fully consistent with the one expected for the modified peptide.

The initial derivatization strategy (strategy 1) for the identification of *s*-nitrosated cysteines by MS only allowed us to identify the cysteine with the highest degree of solvent exposure. To identify non-exposed s-nitrosated cysteines, the derivatization strategy was adapted to unfold the untreated or GSNO-treated CSE with urea prior to the first derivatization step and all throughout the double-labelling procedure (strategy 2 in Supplementary Figure S2). Following this adaptation, we identified a total of seven *s*-nitrosated cysteine residues upon the identification of ions compatible with the tryptic peptides containing Cys84, Cys109, Cys137, Cys172, Cys229, and Cys307/Cys310 with the acrylamide mass increment (Figure 4, Supplementary Figures S4, S6–S10). No signal corresponding to the peptide ^306^QCTGCTGMVTFYIK^319^ with two AA units was observed, which suggests that Cys307 and Cys310 residues are not simultaneously *s*-nitrosated. Nonetheless, the tandem mass spectrum of the double charged ion at *m/z* 840.3854 ± 2.7 ppm, which is compatible with the two isobaric peptides ^306^QCTGCTGMVTFYIK^319^ with the incorporation of AA at one Cys and IAA at the other Cys (^306^QC(IAA)TGC(AA)TGMVTFYIK^319^ and ^306^QC(AA)TGC(IAA)TGMVTFYIK^319^; Supplementary Figure S6), exhibits fragment ions compatible with AA incorporation at Cys307 and Cys310, thereby indicating that both can be *s*-nitrosated. It should be mentioned that regardless of the derivatization strategy, no peptide containing Cys70 was observed by MS, which precluded any conclusion about whether it could also be a site of *s*-nitrosation. Additionally, noteworthy is the fact that all AA-modified Cys residues were also identified as IAA-modified. This can be explained by the lability of the Cys-*s-*NO modification under the derivatization conditions used but can also stem from incomplete *s*-nitrosation of each Cys target.

### 3.6. Functional Analysis of the Serine Variants of Cys70, Cys84, Cys109, Cys137, Cys172, Cys307 and Cys310

Following the strategy adopted to analyze the functional impact of Cys229 *s*-nitrosation, we produced the structurally conservative Cys-to-Ser variants for the other *s*-nitrosation targets identified by MS, namely Cys84, Cys109, Cys137, Cys172, Cys307, and Cys310. In addition, although MS data were uninformative for Cys70, we also produced the C70S variant. The newly generated variants were differently affected by the Cys-to-Ser substitutions in terms of resistance to thermal denaturation compared to WT CSE (Supplementary Figure S5) compared to WT CSE. While C70S, C109S, and C172S showed a slightly increased thermal stability (Δ*T*_m_ ≥ +1 °C), C137S, C307S and C310S exhibited a slight decrease in the melting temperature (Δ*T*_m_ ≤ −1 °C). Overall, the substitutions did not heavily affect the stability of the corresponding variants, which all proved to be remarkably stable proteins, exhibiting *T*_m_ values above 68 °C.

**Figure 5:**
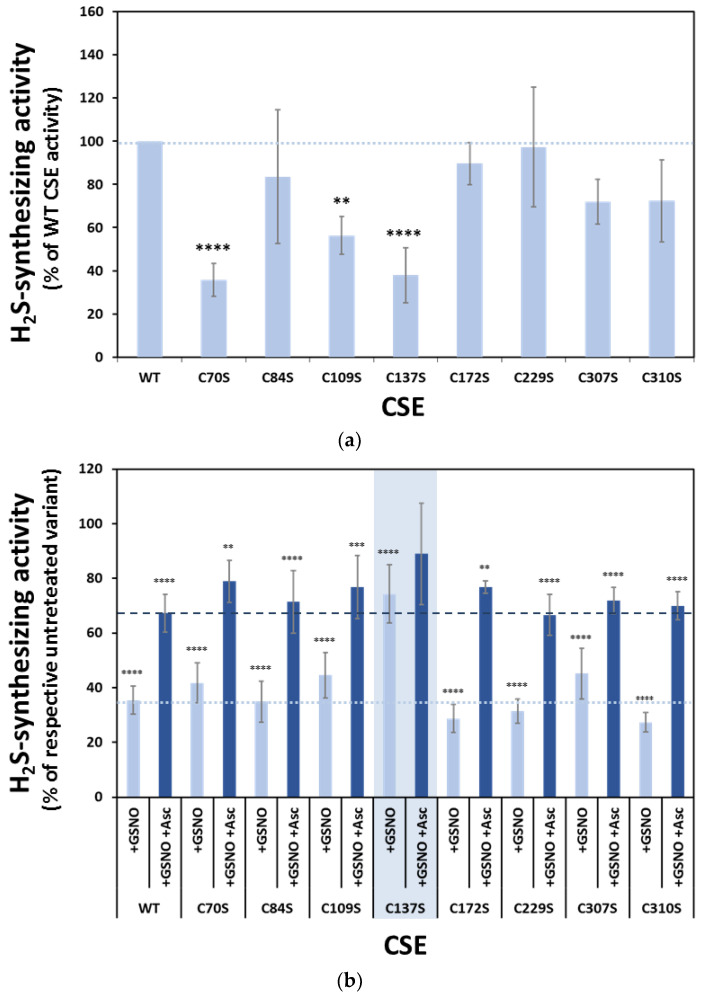
Effect of Cys-to-Ser substitutions on CSE H_2_S-synthesizing activity and GSNO-inhibition. (**a**) Comparison of H_2_S production by untreated WT CSE and Cys-to-Ser variants. Relative activities were measured as described in the legend to Figure 1 and normalized to the WT CSE. Error bars represent the standard error (SE), where *n* ≥ 4. (**b**) Effect of *s*-nitrosation on the enzymatic activity of WT CSE and Cys-to-Ser variants. Histograms represent the relative activities of each variant after GSNO treatment (+GSNO) or the same followed by ascorbate reversal (+GSNO +Asc), normalized to the respective unreacted CSE variant. Error bars represent the standard error (SE), where *n* ≥ 4. Differences between groups were assessed by one-way ANOVA: **** *p* < 0.0001, *** *p* < 0.001, ** *p* < 0.01.

We then evaluated the H_2_S-synthesizing activity of each variant. As shown in Figure 5a, whereas the C84S, C172S, C229S, C307S, and C310S variants exhibited similar activity to WT CSE, the remaining variants were functionally impaired, particularly C70S, C109S, and C137S. Next, we assessed the inhibition of each CSE variant by GSNO (and recovery by ascorbate). We observed that all variants except C137S exhibited essentially WT-like behavior, with activity values of 27–45% in the GSNO-treated compared to the respective untreated variant, and recovery by ascorbate to 67–79% of untreated enzyme activity (Figure 5b). Conversely, the C137S variant appeared remarkably less sensitive to GSNO inhibition, with 74% activity compared to paired untreated samples and non-significant recovery by ascorbate (Figure 5b).

### 3.7. Effect of GSNO on Exposed Cysteines in CSE Cys-to-Ser Variants

**Figure 6:**
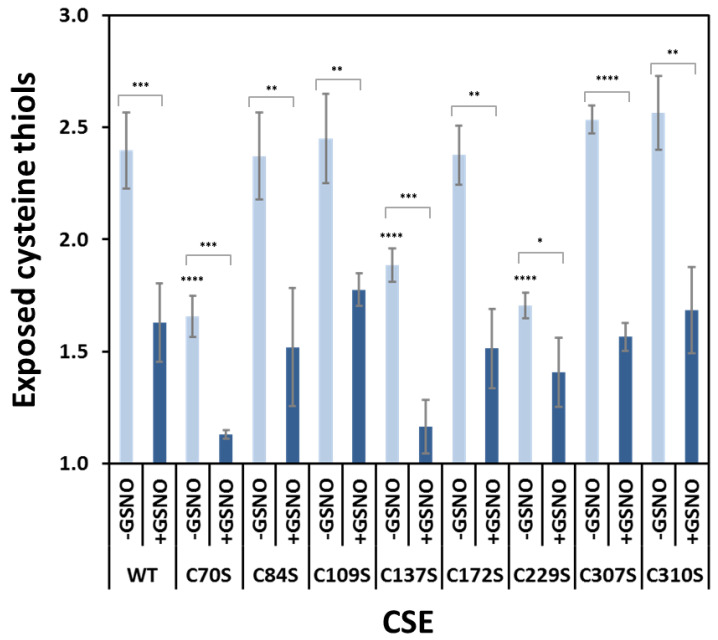
Number of free exposed cysteines as determined using DTNB in WT and mutated CSE for the as isolated protein (-GSNO) and upon incubation with GSNO (+GSNO). Error bars represent the standard error (SE), where *n* ≥ 4. One-way ANOVA was performed to check if there were differences between WT (control) versus the different variants, **** *p* < 0.0001. Student’s *t*-test was used to compare each variant with or without GSNO incubation, **** *p* < 0.0001, *** *p* < 0.001, ** *p* < 0.01, * *p* < 0.05.

To further disclose the identity of the free exposed cysteines in CSE, we employed the DTNB method to analyze the remainder of the Cys-to-Ser variants. As shown in Figure 6, whereas the C84S, C109S, C172S, C307S, and C310S variants exhibited the same number of exposed cysteines as the WT CSE, C70S, and C137S displayed similar values to C229S (i.e., approximately one cysteine less than the WT protein). Moreover, upon incubation with GSNO, all variants showed less exposed cysteine thiols to various degrees. While C229S decreased only from 1.7 to 1.4 exposed thiols upon GSNO treatment, C70S and C137S exhibited larger decreases of 0.5 and 0.7 exposed thiols, respectively.

## 4. Discussion

Despite growing interest in the role of gasotransmitters in human pathophysiology, the regulatory networks that tightly control the levels of these reactive species in different physiological contexts are slowly but surely unravelled. A body of evidence accumulated from studies on different models from isolated proteins to cellular and animal models point to a cross-regulation between the three gasotransmitters, whereby they control each other’s levels. While NO and CO have long been reported to inhibit CBS-derived H_2_S production, thereby affecting different cellular pathways, the effect of these gasotransmitters on CSE function and structure has thus far been underappreciated.

Herein, we combined functional assays with biochemical and biophysical analytical tools to investigate the effect of *s*-nitrosation on H_2_S generation by human CSE. Human CSE has ten non-disulfide cysteine residues, an unusually high number of such residues. Upon incubation with the physiological *s*-nitrosating agent *s*-nitrosoglutathione (GSNO), CSE activity was inhibited by ≈70% (Figure 1). Interestingly, the selective CSE inhibitor propargylglycine maximally inhibits CSE to a similar extent (20–30% residual activity), similar to the NO releaser diethylamine NONOate [39,44]. Restoring CSE activity upon reduction in the putatively *s*-nitrosated cysteine(s) with excess sodium ascorbate resulted in partial functional recovery (Figure 1). Notably, although GSNO alone did not seem to affect the PLP spectral features, the *s*-nitrosation reversal in the presence of large excess of ascorbate slightly decreased the cofactor load consistently with the incomplete activity recovery. As control experiments, we ruled out the effects of the sole ascorbate or reduced glutathione on CSE function, pointing to cysteine *s*-nitrosation as responsible for CSE inhibition.

**Figure 7:**
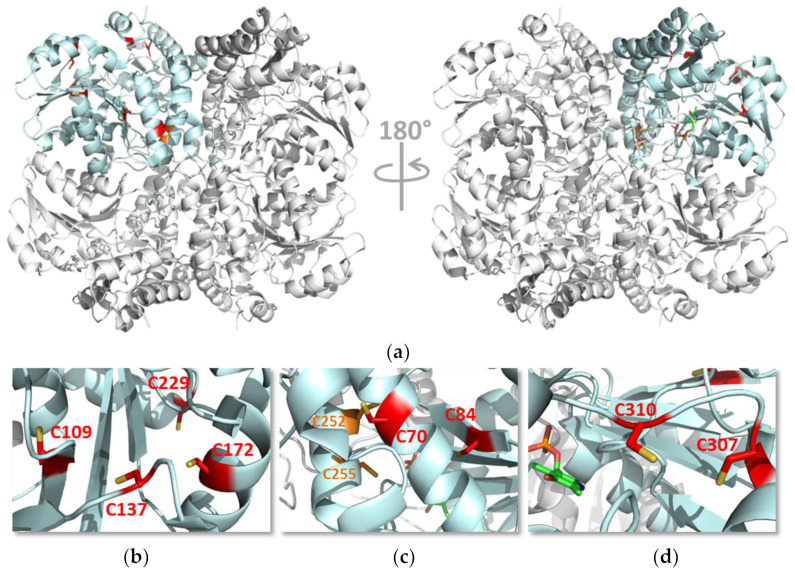
Structure of human cystathionine γ-lyase. (**a**) Cartoon representation of the crystallographic structure of human CSE (PDB ID: 2NMP). One monomer is colored in light blue, while the remaining monomers are shown in grey. (**b**–**d**) Zoom-in on the regions of the protein where the cysteine residues investigated in this study are located. Red sticks, Cys residues that have been shown to be *s*-nitrosated; orange sticks, Cys residues without evidence for *s*-nitrosation; green sticks, PLP moiety in the active site.

To identify the cysteine residues targeted by *s*-nitrosation with a functional impact on enzymatic activity, we used MS-based analysis upon labelling with two different derivatization agents. Using the protein derivatization strategy 1 (Supplementary Figure S1), Cys229 was identified as the single solvent-exposed cysteine residue to be *s*-nitrosated (Figure 2). We generated the C229S variant to assess the relevance of this residue and observed that it exhibited a similar enzymatic activity as the WT CSE, displaying the same degree of inhibition by GSNO (Figure 3). Consistent with the predicted exposure of Cys229 based on CSE crystallographic structure (Figure 7, Supplementary Table S1 and Supplementary Figure S11), the number of exposed thiols in C229S significantly decreased with respect to the WT CSE, and further exhibited a small decrease upon GSNO treatment (Figure 3). Given that the MS-based dual derivatization strategy initially undertaken could have overlooked the buried *s*-nitrosated cysteine residues, we modified the derivatization procedure (Strategy 2 in Supplementary Figure S1). By chemically unfolding the untreated or *s*-nitrosated WT CSE with urea prior to derivatization, we exposed all surface-exposed and -occluded cysteine residues. This allowed us to detect a total of seven acrylamide-derivatized cysteines, thereby identified as *s*-nitrosation targets (Figure 4). This striking difference between the two dual derivatization strategies highlights the difficulties in the assignment of cysteine residues as targets of post-translational modifications, particularly oxidative ones (reviewed e.g., in [57]).

Based on the newly revealed target cysteine residues, we generated conservative Cys-to-Ser variants of each of those residues including Cys70, which could still have been modified despite no MS data were obtained. The introduced amino acid substitutions had a mild impact on protein stability, slightly destabilizing some variants or stabilizing others. Despite the structural and functional relevance of cysteine residues, protein engineering strategies for stabilization of recombinant proteins often involves the substitution of cysteine residues by a relatively structurally conservative serine. Regardless of the structural impact, from the eight Cys-to-Ser variants only three were clearly functionally impaired compared to WT CSE, namely C70S, C109S, and C137S, exhibiting 36–56% of the WT enzymatic activity (Figure 5). The remaining displayed enzymatic activities similar to the WT enzyme (Figure 5). These results only partially overlap with those reported by Luo et al. for the corresponding Cys-to-Ala substitutions [23]. In that study, C84A, C109A, and C307A CSE had statistically significant lower activity than the WT enzyme, whereas the C70A variant had 50% higher activity. These differences highlight the caution required when generating protein variants with cysteine substitutions, since the size and polarity of the side chain can be relevant aside from the thiol chemistry. Herein, despite the C70S, C109S, and C137S variants being functionally impaired, only C137S revealed to be relatively insensitive to GSNO-derived inhibition, whereas all other variants exhibited the same degree of GSNO sensitivity as the WT enzyme (Figure 5). This observation points to Cys137 as the main target residue of CSE *s*-nitrosation contributing to GSNO-mediated inhibition. To further understand the functional and structural impact of *s*-nitrosation, we analyzed the number of exposed thiols in each variant by the DTNB assay and observed WT-like patterns for all variants, except C70S, C137S, and, as mentioned above, C229S (Figure 6). This further confirms that three free exposed cysteine residues are present in the WT CSE, consistent with the 2.5 exposed thiols quantitated by DTNB in the WT CSE. This observation only partially overlaps with the solvent accessibility calculated based on the crystallographic structure (Supplementary Table S1 and Supplementary Figure S11), which predicts Cys70, Cys84, Cys229, and Cys307 to be exposed. However, a close inspection of each residue revealed the Cys84 side chain to be turned inward toward the core of the protein. Moreover, while the Cys307 side chain appeared to be partially exposed, it is likely secluded from the solvent by the missing N-terminal region that is not present in the available crystallographic structure. In contrast, whereas Cys137 was not predicted to be exposed, the region where it is located has a high degree of flexibility, as observed by the B-factor representation (Supplementary Figure S12). Therefore, this increased mobility and flexibility is likely to contribute to expose Cys137, which could not have been predicted from the static crystallographic structure. Besides the C70S, C137S and C229S variants allowing us to identify the CSE solvent exposed cysteines, their GSNO treatment resulted in a further decrease in exposed thiols to different degrees, which suggests partial *s*-nitrosation of each of these residues. This is consistent with the MS analysis, which always revealed a mixture of acrylamide- and iodoacetamide-derivatized cysteines for these residues upon GSNO treatment. However, we cannot rule out that such a mixture arises from the lability of *s*-nitrosated cysteines and the stringent chemical treatment inherent to the analysis. Regardless, among the exposed cysteine residues, Cys229 emerged as the main target of *s*-nitrosation, since GSNO treatment of the C229S variant led to the smallest decrease in exposed thiols as detected by the DTNB assay (Figure 6).

We sought to understand the impact of the cysteine residues on CSE structure and function, particularly those that seem functionally relevant and/or affording GSNO-mediated inhibition, by inspecting the CSE crystallographic structure (Figure 7 and Supplementary Figures S11 and S12) and the sequence conservation of these residues (Supplementary Figure S13). Cys70 is structurally located in the same helix as Thr67, which appears to be relevant for enzymatic activity, as the T67I variant identified in cystathioninuria patient(s) is reported to display significantly reduced enzymatic activity compared to the WT CSE [10,44]. Cys109 precedes the mobile loop region between Met110 and Asn118, which forms one of the loops flanking the PLP binding cleft in the active site. Moreover, in this loop, the aromatic side chain of the Tyr114 region displays π-stacking interactions with the pyridoxine ring of PLP. Cys137 is also located in a highly mobile region very close to this same loop (Supplementary Figure S12). It is thus plausible that substitution of Cys109 or Cys137 by serine structurally affects this region near the active site with functional consequences. Despite the functional impairment of the serine variants of these cysteine residues, only Cys137 appears to be relevant for GSNO-mediated inhibition. Again, *s*-nitrosation of this residue may have an impact on the mobile loops flanking the active site. Notably, similar to most cysteine residues in CSE, Cys137 is conserved in mammalian CSE, while it is absent in other eukaryotes. Conversely, Cys229 is the only completely non-conserved Cys residue in CSE, being only present in the human enzyme. Thus far, no consensus motif is known to fully and unequivocally predict *s*-nitrosation sites. However, the environment surrounding a particular cysteine residue likely determines its predisposition for *s*-nitrosation. Flanking acidic/basic residues, distally located charged residues, low pKa, nearby hydrophobicity, α-helical location, and a large solvent-accessible area are common to *s*-nitrosatable cysteines [41,43]. Oddly, the protein environment surrounding Cys137 residue in human CSE appears to be mostly hydrophobic. However, this information is based solely on the ‘static’ crystallographic structures, which may fail to reveal the different microenvironments the Cys137 side chain could probe, given the high flexibility of the region where it is embedded.

In conclusion, while protein-mediated control of NO and CO availability by H_2_S has been thoroughly investigated and documented (reviewed e.g., in [1]), the evidence for NO- and CO-mediated modulation of H_2_S levels is still accumulating. Most studies on this topic concern either the direct reactions between reactive nitrogen species and reactive sulfide species (reviewed e.g., in [24,25]), or the inhibition of human CBS by NO and CO (reviewed e.g., in [5,8,34]). The latter has been thoroughly characterized, and different cellular and physiological consequences of this regulatory mechanism have been demonstrated [8,9,59,60,61]). While H_2_S has been reported to affect protein *s*-nitrosation (e.g., in [62]), the discovery of inhibition of human CSE upon *s*-nitrosation unravels another mechanism of crosstalk between NO and H_2_S with various predictable consequences in terms of human physiology and pathophysiology. Moreover, this regulatory crosstalk centered at human CSE offers the perspective of drug development for various human diseases including cancer (reviewed e.g., in [5,63]).

## 5. Conclusions

In human (patho)physiology, cross-regulation of each ‘gasotransmitter’ by one another affords an intricate web of regulatory mechanisms that ensures an effective signaling function at safe homeostatic levels. Regulation of CSE-mediated H_2_S production via protein *s*-nitrosation affords an extra layer of complexity to this network and offers another possible avenue of pharmacological modulation of H_2_S availability for pathologies associated with disturbed H_2_S metabolism.

## Data Availability

The mass spectrometry datasets analyzed for this study can be found in the ProteomeXchange Consortium via the PRIDE partner repository. All other data is contained within the article and Supplementary Materials.

## Supplementary Materials

The following are available online at https://www.mdpi.com/article/10.3390/antiox10091391/s1, Figure S1: Dual derivatization strategies; Figure S2: Dual derivatization protocols; Figure S3: Spectral features of human CSE PLP cofactor upon incubation with GSNO; Figure S4: MS/MS spectrum confirming acrylamide incorporation at Cys229; Figure S5: Resistance to thermal denaturation of CSE variants analyzed by dye-free differential scanning fluorimetry; Figures S6–S10: MS/MS spectra confirming acrylamide incorporation at Cys307 (S6), Cys310 (S6), Cys84 (S7), Cys109 (S8), Cys137 (S9), and Cys172 (S10); Figure S11: Solvent-accessibility of cysteine residues in CSE; Figure S12: Structural flexibility of CSE; Figure S13: Sequence alignment of CSE homologues; Supplementary Table S1: Assessment of cysteine surface accessibility; Sequences of CSE variants inserted in pET28b between the NcoI and BamHI sites (underlined in each sequence).
