# Supplementary material for: Human Cystathionine γ-Lyase Is Inhibited by s-Nitrosation: A New Crosstalk Mechanism between NO and H2S
Source: Antioxidants (Basel). 2021 Aug 30;10(9):1391. doi: 10.3390/antiox10091391 (PMC8467691; doi:10.3390/antiox10091391)
Supplement: Supplementary file 1 [file antioxidants-10-01391-s001.zip › antioxidants-1335694-supplementary.pdf]

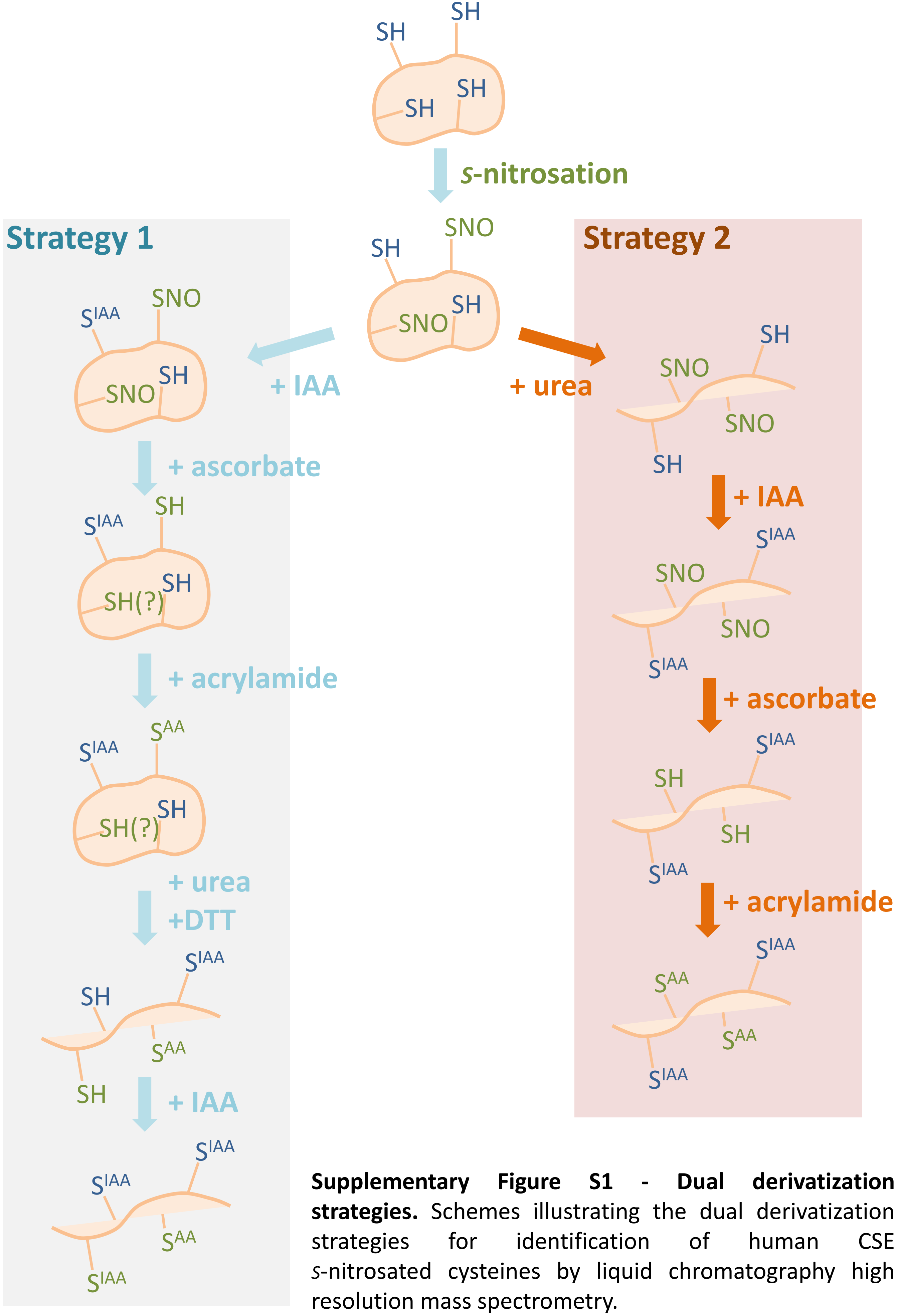

Supplementary Figure S1 - Dual derivatization strategies. Schemes illustrating the dual derivatization strategies for identification of human CSE *s*-nitrosated cysteines by liquid chromatography high resolution mass spectrometry.

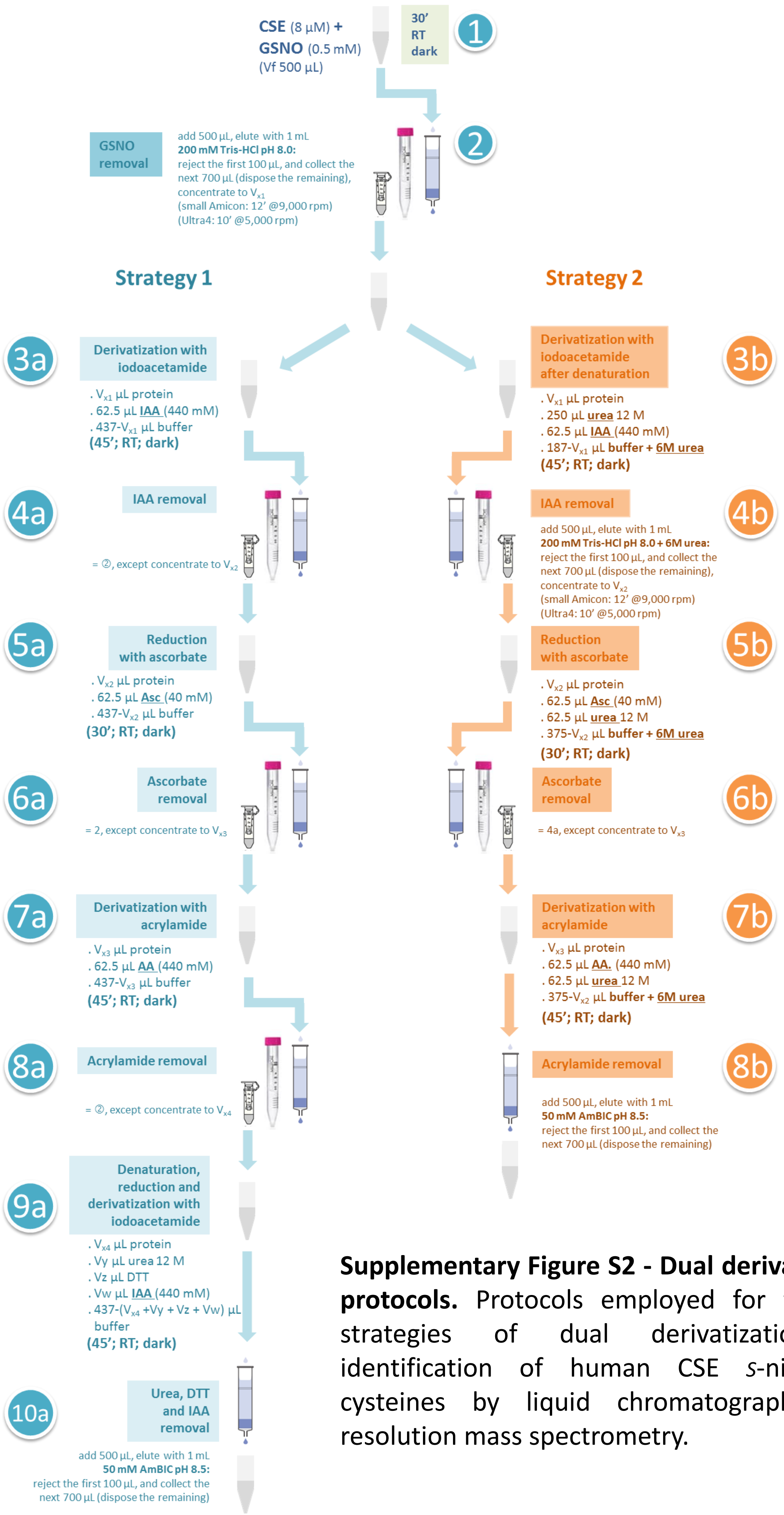

Supplementary Figure S2 - Dual derivatization protocols. Protocols employed for the two strategies of dual derivatization for identification of human CSE *s*-nitrosated cysteines by liquid chromatography high resolution mass spectrometry.

Supplementary Figure S2 **Fernandes et al (2021)**

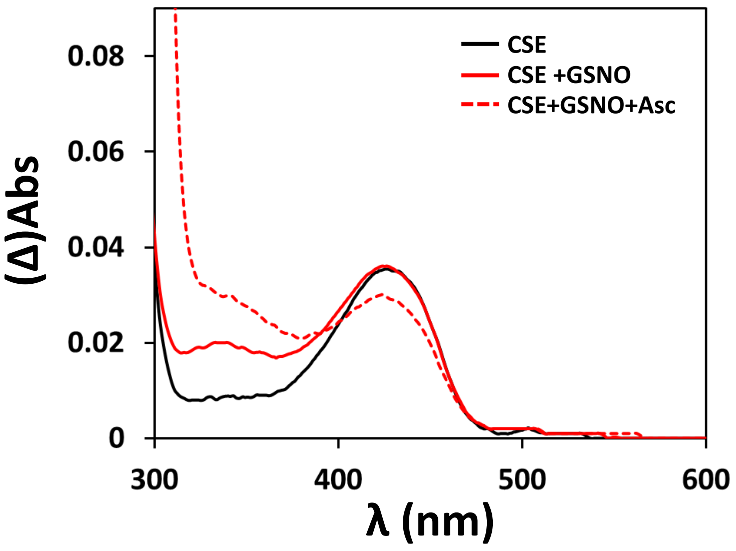

Supplementary Figure S3 - Spectral features of human CSE PLP cofactor upon incubation with GSNO. UV-visible of recombinant CSE (6 µM, in 200 mM Tris-HCl, pH 8.0) collected for the unreacted protein (solid black line), for CSE incubated with 40 µM for 30 minutes (solid red line), and upon addition of ascorbate to the latter sample and incubation for 30 minutes (dashed red line).

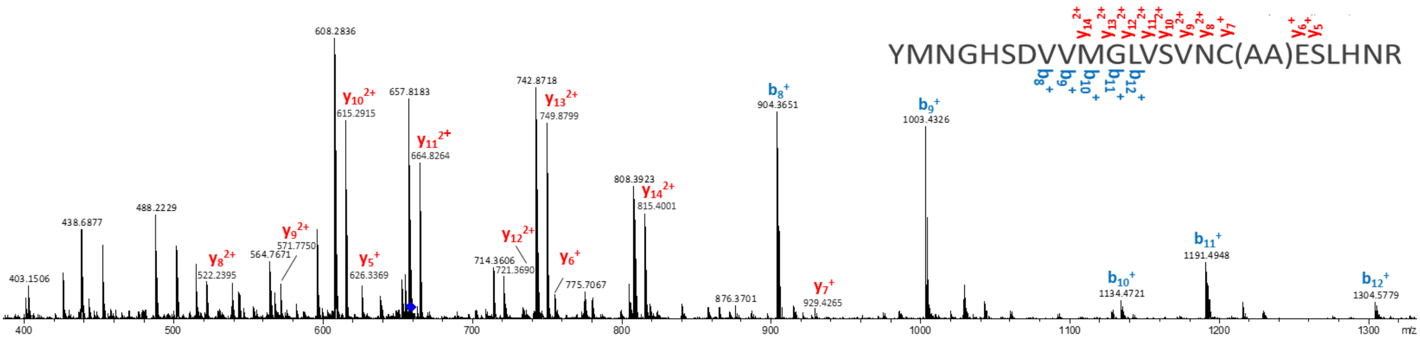

Supplementary Figure S4 - MS/MS spectrum of the double charged ion at *m/z* 658.8111 ± 6.1 ppm, corresponding to the tryptic CSE peptide [213]YMNGHSDVVMGLVSVNCESLHNR[235] with acrylamide (AA) incorporation at the Cys229 residue. Labeled in red are y ions and in blue are b ions.

Supplementary Figure S4 **Fernandes et al (2021)**

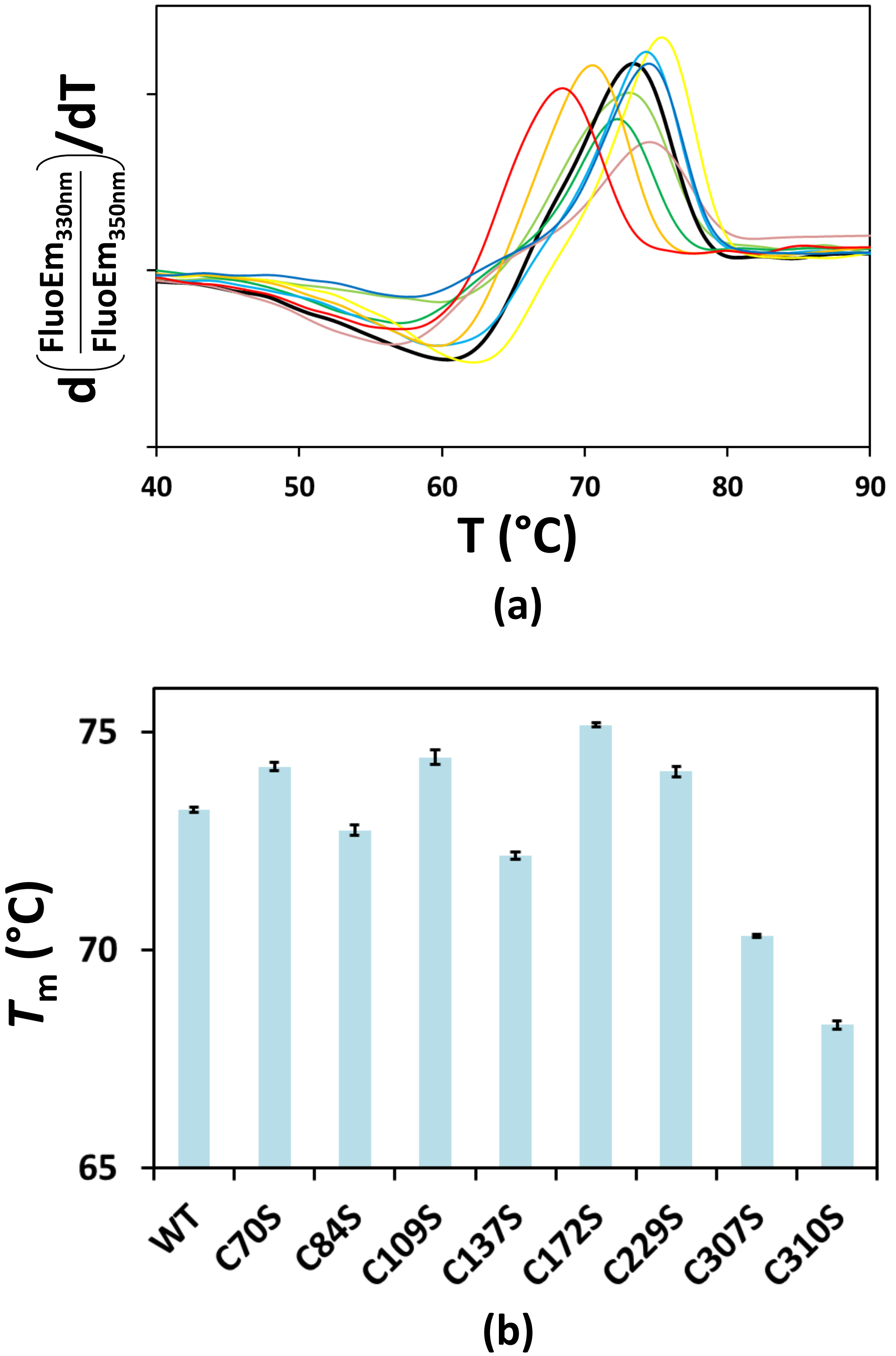

Supplementary Figure S5 - Resistance to thermal denaturation of CSE variants analyzed by dye-free differential scanning fluorimetry. (a) first derivative of the thermal denaturation profiles given from the ratio between emitted fluorescence at 330 nm and 350 nm as a function of temperature. Each curve represents the average of three replicates. black - WT, dark blue - C70S, light green - C84S, pink - C109S, dark green - C137S, yellow - C172S, light blue - C229S, orange - C307S, red - C310S **(b)** Melting temperatures (T_m) calculated from the peaks of the first derivative curves in (a). Data are presented as the average of three replicates.

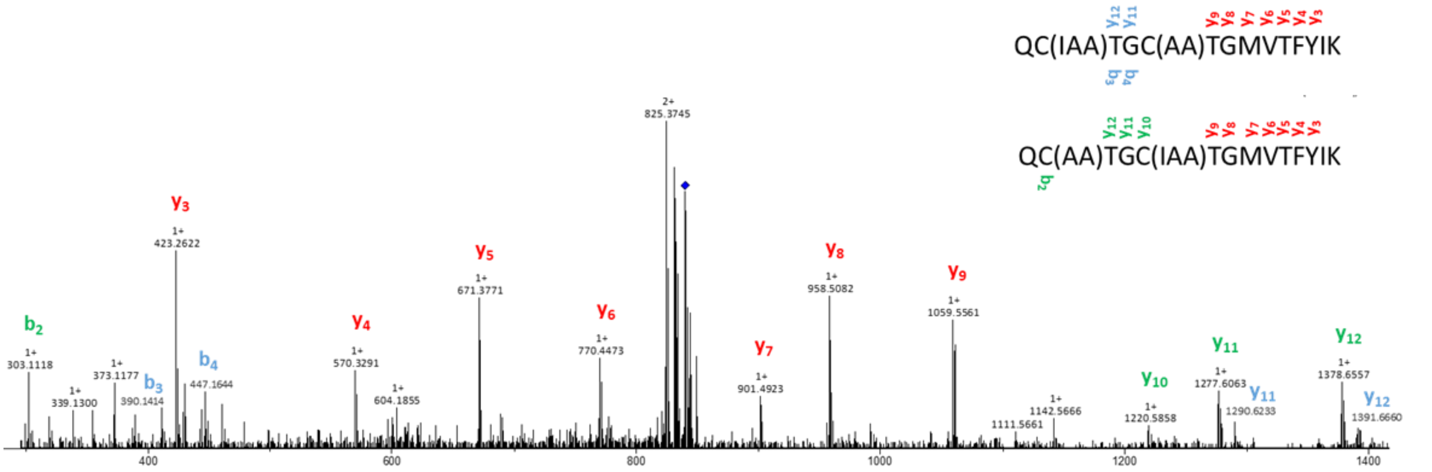

Supplementary Figure S5 - MS/MS spectrum of the double charged ion at *m/z* 840.3854 ± 2.7 ppm, corresponding to the two isobaric ^{306}QCTGCTGMVTFYIK^{319} peptides with incorporation of acrylamide (AA) at one Cys and iodoacetamide (IAA) at the other Cys residue. Labelled in green are ions compatible with Cys307 *s*-nitrosation: b_2^+ ion at *m/z* 303.1118 and y_{12}^+, y_{11}^+ and y_{10}^+ ions (at *m/z* 1378.6557, 1277.6063 and 1220.5858, respectively) of ^{306}QC(AA)TGC(IAA)TGMVTFYIK^{319} peptide. Labelled in blue are ions compatible with Cys310 *s*-nitrosation: y_{12}^+ and y_{11}^+ (at *m/z 1391.6660 and 1290.6233,* respectively) and b_4^+ and b_3^+ ions (at *m/z* 447.1644 and 390.1414, respectively) of peptide ^{306}QC(IAA)TGC(AA)TGMVTFYIK^{319}. Labeled in red are the ions that are common to both peptides.

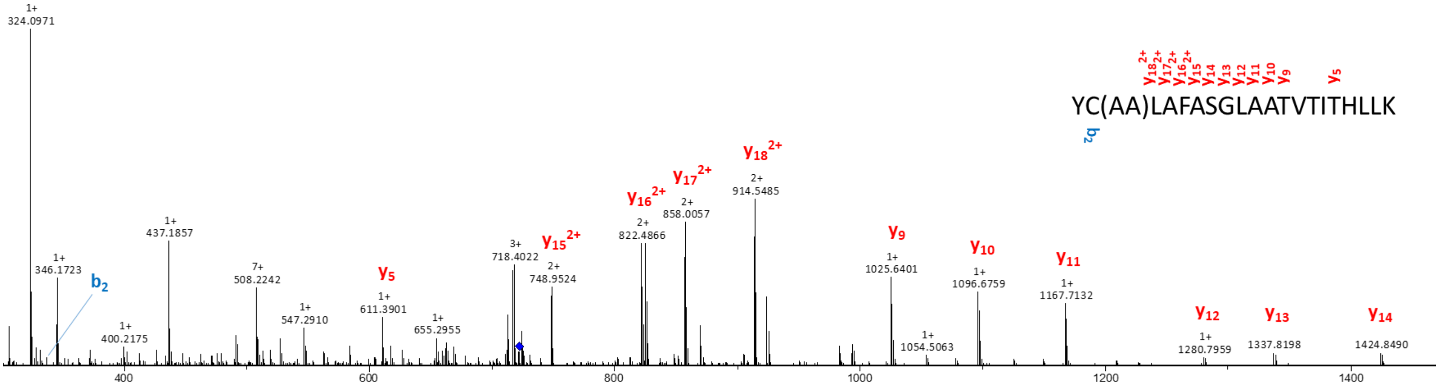

Supplementary Figure S6 - MS/MS spectrum of the triple charged ion at *m/z* 722.0686 ± 3.6 ppm, corresponding to the tryptic CSE peptide ^{83}YCLAFASGLAATVTITHLLK^{102} with acrylamide (AA) incorporation at the Cys84 residue. Labeled in red are y ions and in blue are b ions.

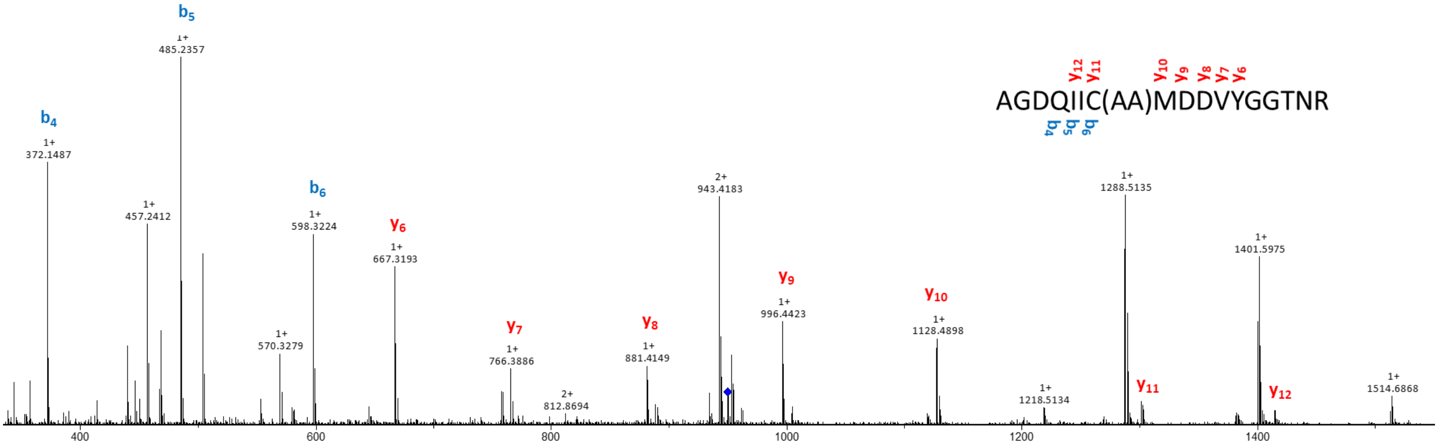

Supplementary Figure S8 - MS/MS spectrum of the double charged ion at *m/z* 949.9240 ± 1.9 ppm, corresponding to the tryptic CSE peptide 103AGDQIICMDDVYGGTNR119 with acrylamide (AA) incorporation at the Cys109 residue. Labeled in red are y ions and in blue are b ions.

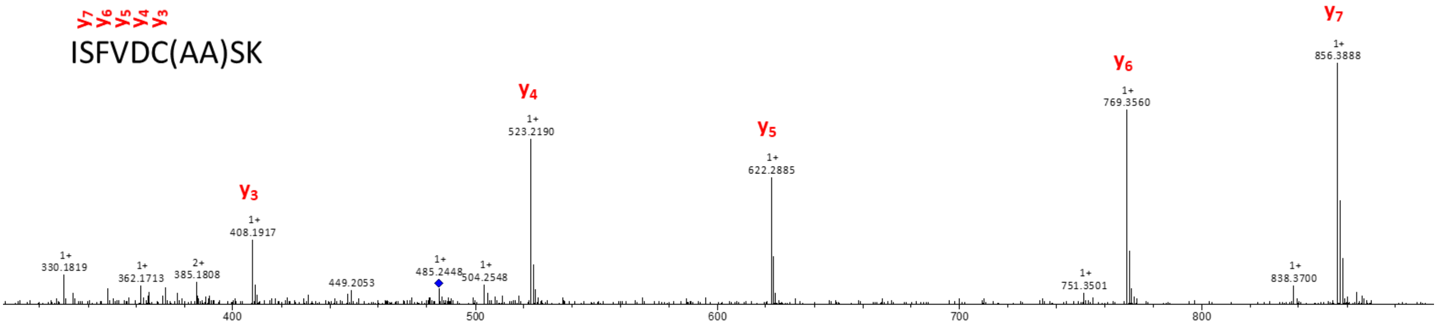

Supplementary Figure S9 - MS/MS spectrum of the double charged ion at *m/z* 485.2410 ± 3.7 ppm, corresponding to the tryptic CSE peptide 132ISFVDCSK139 with acrylamide (AA) incorporation at the Cys137 residue. Labeled in red are y ions.

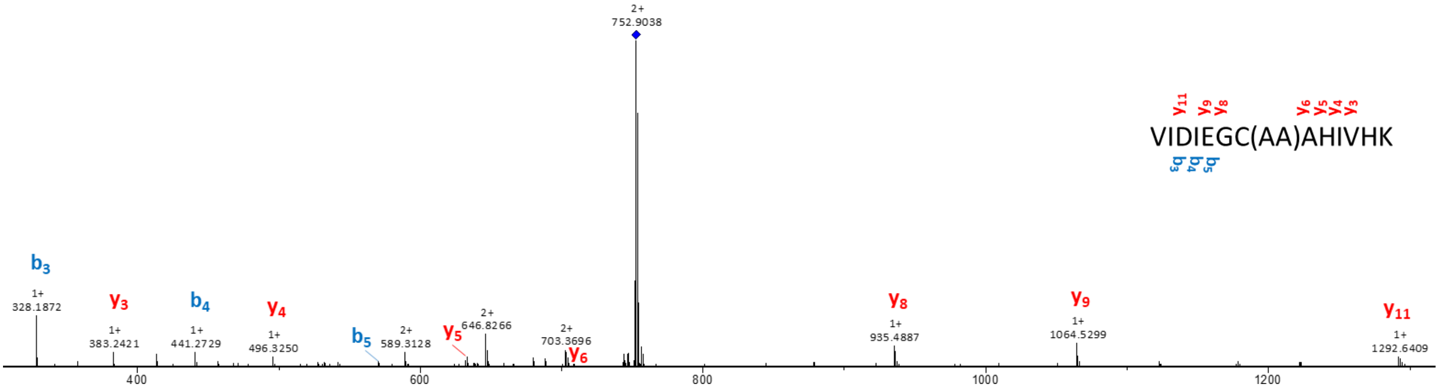

Supplementary Figure S10 - MS/MS spectrum of the triple charged ion at *m/z* 752.9038 ± 4.1 ppm, corresponding to the tryptic CSE peptide 166VIDIEGCAHIVHK178 with acrylamide (AA) incorporation at the Cys172 residue. Labeled in red are y ions and in blue are b ions.

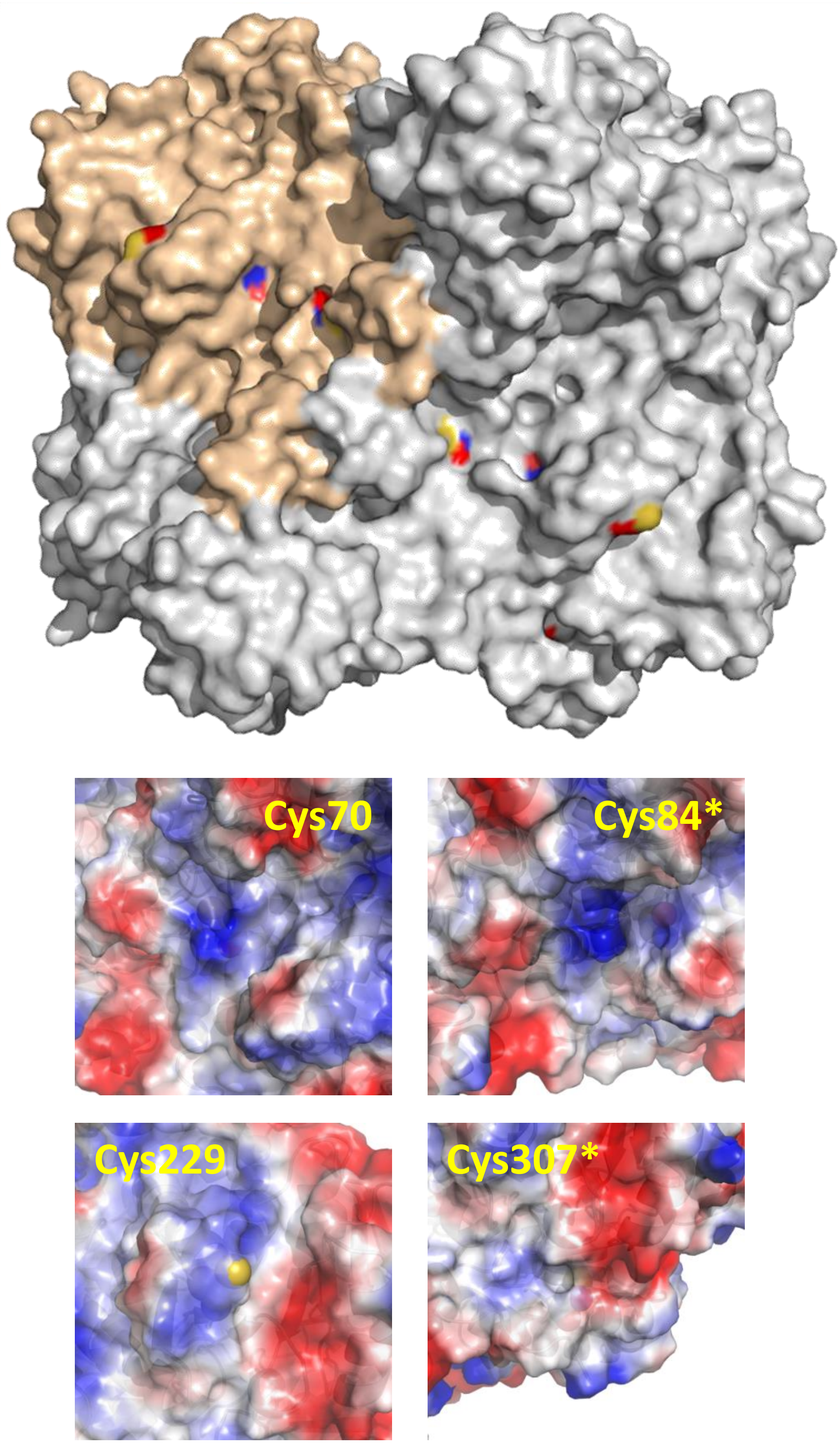

Supplementary Figure S11 - Solvent-accessibility of cysteine residues in CSE. The quaternary structure of tetrameric human CSE is represented in surface mode (one monomer colored in pale orange). Cysteine residues are highlighted in red in the first subunit. Four cysteines (Cys70, Cys84, Cys229, Cys307) per subunit are calculated to be solvent accessible. * indicates the cysteines that, despite being solvent accessible, have the thiol group pointing towards the inside of the protein. Coordinates correspond to PBD entry 6NBA. Figure generated with Pymol.

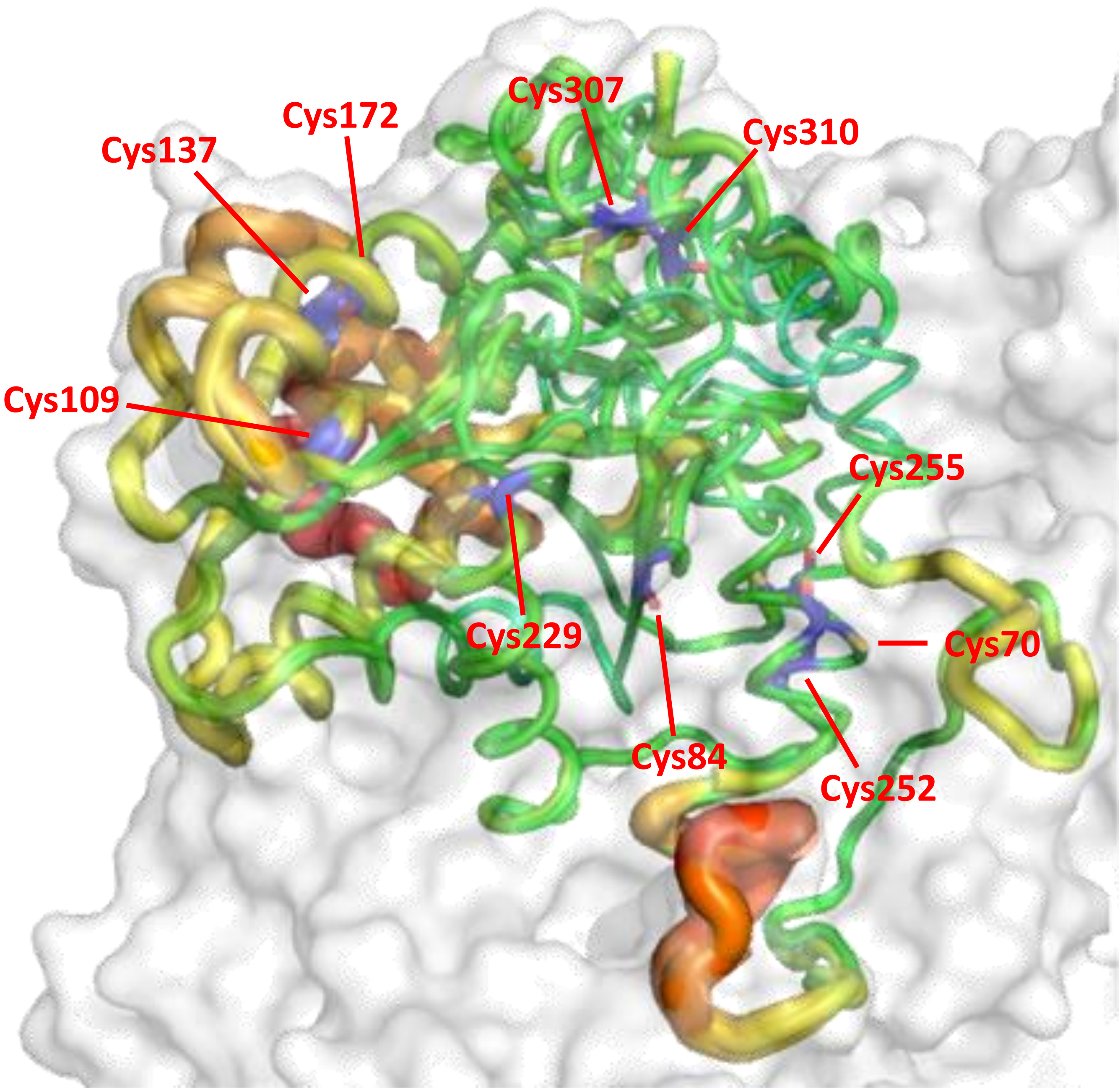

Supplementary Figure S12 - Structural flexibility of CSE. Cartoon representation of the flexibility (B factors of the main chain) of the structure of human CSE showing the positions of all cysteine residues (as blue sticks). The four chains (A, B, C and D) of PDB entry 2NMP are aligned and overlapped. Flexibility is represented both by the thickness and the color code of the main chain: flexibility is directly proportional to the thickness and increases along cyan<green<yellow<orange<red. Figure generated with Pymol.

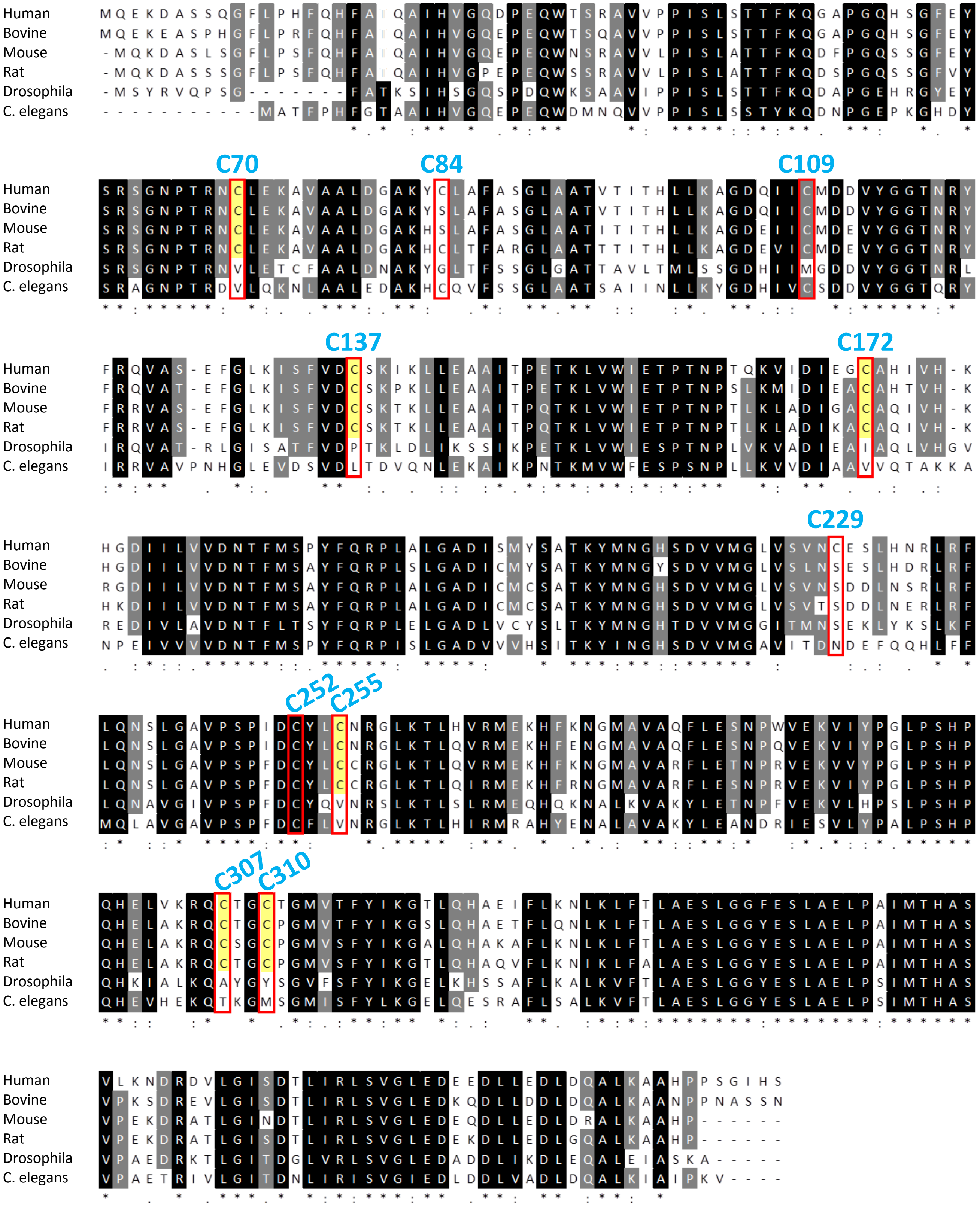

Supplementary Figure S13 - Sequence alignment of CSE homologues. The sequences of human (P32929-1), *Bos taurus* (bovine; Q58DW2), *Mus musculus* (Mouse; Q8VCN5), *Rattus norvegicus* (Rat; P18757), *Drosophila melanogaster* (Drosophila; Q7JXZ2), and *Caenorhabditis elegans* (C. elegans; P55216) CSE were aligned with ClustalX.

PDB ID	Chain ID	Accessible surface area (Å^2) — Cysteine residue 70	84	109	137	172	229	252	255	307	310
6NBA	A	3.30	2.45	0.00	0.12	0.50	69.39	0.00	0.00	3.31	0.33
	B	3.96	2.46	0.00	0.00	0.00	67.63	0.00	0.00	3.64	0.00
	C	6.65	2.32	0.00	0.00	0.00	69.01	0.00	0.00	3.31	0.33
	D	3.80	2.16	0.00	0.00	0.67	67.73	0.00	0.00	3.48	1.01
2NMP	A	3.42	3.80	0.00	0.00	0.33	63.21	0.16	0.00	3.81	0.67
	B	3.63	2.74	0.00	2.09	0.66	69.07	0.00	0.00	3.47	1.51
	C	2.16	3.55	0.00	0.86	0.33	63.92	0.00	0.00	3.97	1.33
	D	1.95	3.72	0.00	0.12	0.50	63.60	0.17	0.00	3.97	1.01

Supplementary Table S1. Assessment of cysteine surface accessibility. Each monomer of human CSE contains a total of ten cysteine residues. To estimate the solvent-accessibility of these cysteines, we analyzed the accessible surface area of each residue from the crystallographic structures available at the Protein Data Bank (PDB) using the PDBe PISA tool [E. Krissinel and K. Henrick (2007) 'Inference of macromolecular assemblies from crystalline state.' J. Mol. Biol. 372, 774-797]. Cys229 shows the largest accessible surface area (≈67 Å^2), followed by Cys70, Cys84 and Cys307 (≈3 Å^2) (Supplementary figure S11). The six remaining cysteine residues do not appear to be surface-exposed.

Sequences of CSE variants inserted in pET28b between the NcoI and BamHI sites (underlined in each sequence).

>CSE_WT
ccatgggcCATATGCACCATCATCATCATCATTCTTCTGGTGTAGATCTGGGTACCGAGAACCTGTACTTCCAATCCATGCAGGAAAAAGACGCCTCCTCACAAGGTTTCC
TGCCACACTTCCAACATTTCGCCACGCAGGCGATCCATGTGGGCCAGGATCCAGAGCAATGGACCTCCAGGGCTGTAGTGCCCCCCATCTCACTGTCCACCACGTTCAAGC
AAGGGGCGCCTGGCCAGCACTCGGGTTTTGAATATAGCCGTTCTGGAAATCCCACTAGGAATTGCCTTGAAAAAGCAGTGGCAGCACTGGATGGGGCTAAGTACTGTTTGG
CCTTTGCTTCAGGTTTAGCAGCCACTGTAACTATTACCCATCTTTTAAAAGCAGGAGACCAAATTATTTGTATGGATGATGTGTATGGAGGTACAAACAGGTACTTCAGGC
AAGTGGCATCTGAATTTGGATTAAAGATTTCTTTTGTTGATTGTTCCAAAATCAAATTACTAGAGGCAGCAATTACACCAGAAACCAAGCTTGTTTGGATCGAAACCCCCA
CAAACCCCACCCAGAAGGTGATTGACATTGAAGGCTGTGCACATATTGTCCATAAGCATGGAGACATTATTTTGGTCGTGGATAACACTTTTATGTCACCATATTTCCAGC
GCCCTTTGGCTCTGGGAGCTGATATTTCTATGTATTCTGCAACAAAATACATGAATGGCCACAGTGATGTTGTAATGGGCCTGGTGTCTGTTAATTGTGAAAGCCTTCATA
ATAGACTTCGTTTCTTGCAAAACTCTCTTGGAGCAGTTCCATCTCCTATTGATTGTTACCTCTGCAATCGAGGTCTGAAGACTCTACATGTCCGAATGGAAAAGCATTTCA
AAAACGGAATGGCAGTTGCCCAGTTCCTGGAATCTAATCCTTGGGTAGAAAAGGTTATTTATCCTGGGCTGCCCTCTCATCCACAGCATGAGTTGGTGAAGCGTCAGTGTA
CAGGTTGTACAGGGATGGTCACCTTTTATATTAAGGGCACTCTTCAGCATGCTGAGATTTTCCTCAAGAACCTAAAGCTATTTACTCTGGCCGAGAGCTTGGGAGGATTCG
AAAGCCTTGCTGAGCTTCCGGCAATCATGACTCATGCATCAGTTCTTAAGAATGACAGAGATGTCCTTGGAATTAGTGACACACTGATTCGACTTTCTGTGGGCTTAGAGG
ATGAGGAAGACCTACTGGAAGATCTAGATCAAGCTTTGAAGGCAGCACACCCTCCAAGTGGATGACAGTAAAGGTGGATACGGATCCGAATAATAATAA ctcgag

>CSE_C70S
ccatgggcCATATGCACCATCATCATCATCATTCTTCTGGTGTAGATCTGGGTACCGAGAACCTGTACTTCCAATCCATGCAGGAAAAAGACGCCTCCTCACAAGGTTTCC
TGCCACACTTCCAACATTTCGCCACGCAGGCGATCCATGTGGGCCAGGATCCAGAGCAATGGACCTCCAGGGCTGTAGTGCCCCCCATCTCACTGTCCACCACGTTCAAGC
AAGGGGCGCCTGGCCAGCACTCGGGTTTTGAATATAGCCGTTCTGGAAATCCCACTAGGAATAGCCTTGAAAAAGCAGTGGCAGCACTGGATGGGGCTAAGTACTGTTTGG
CCTTTGCTTCAGGTTTAGCAGCCACTGTAACTATTACCCATCTTTTAAAAGCAGGAGACCAAATTATTTGTATGGATGATGTGTATGGAGGTACAAACAGGTACTTCAGGC
AAGTGGCATCTGAATTTGGATTAAAGATTTCTTTTGTTGATTGTTCCAAAATCAAATTACTAGAGGCAGCAATTACACCAGAAACCAAGCTTGTTTGGATCGAAACCCCCA
CAAACCCCACCCAGAAGGTGATTGACATTGAAGGCTGTGCACATATTGTCCATAAGCATGGAGACATTATTTTGGTCGTGGATAACACTTTTATGTCACCATATTTCCAGC
GCCCTTTGGCTCTGGGAGCTGATATTTCTATGTATTCTGCAACAAAATACATGAATGGCCACAGTGATGTTGTAATGGGCCTGGTGTCTGTTAATTGTGAAAGCCTTCATA
ATAGACTTCGTTTCTTGCAAAACTCTCTTGGAGCAGTTCCATCTCCTATTGATTGTTACCTCTGCAATCGAGGTCTGAAGACTCTACATGTCCGAATGGAAAAGCATTTCA
AAAACGGAATGGCAGTTGCCCAGTTCCTGGAATCTAATCCTTGGGTAGAAAAGGTTATTTATCCTGGGCTGCCCTCTCATCCACAGCATGAGTTGGTGAAGCGTCAGTGTA
CAGGTTGTACAGGGATGGTCACCTTTTATATTAAGGGCACTCTTCAGCATGCTGAGATTTTCCTCAAGAACCTAAAGCTATTTACTCTGGCCGAGAGCTTGGGAGGATTCG
AAAGCCTTGCTGAGCTTCCGGCAATCATGACTCATGCATCAGTTCTTAAGAATGACAGAGATGTCCTTGGAATTAGTGACACACTGATTCGACTTTCTGTGGGCTTAGAGG
ATGAGGAAGACCTACTGGAAGATCTAGATCAAGCTTTGAAGGCAGCACACCCTCCAAGTGGATGACAGTAAAGGTGGATACGGATCCGAATAATAATAA ctcgag

>CSE_C84S
ccatgggcCATATGCACCATCATCATCATCATTCTTCTGGTGTAGATCTGGGTACCGAGAACCTGTACTTCCAATCCATGCAGGAAAAAGACGCCTCCTCACAAGGTTTCC
TGCCACACTTCCAACATTTCGCCACGCAGGCGATCCATGTGGGCCAGGATCCAGAGCAATGGACCTCCAGGGCTGTAGTGCCCCCCATCTCACTGTCCACCACGTTCAAGC
AAGGGGCGCCTGGCCAGCACTCGGGTTTTGAATATAGCCGTTCTGGAAATCCCACTAGGAATTGCCTTGAAAAAGCAGTGGCAGCACTGGATGGGGCTAAGTACtccTTGG
CCTTTGCTTCAGGTTTAGCAGCCACTGTAACTATTACCCATCTTTTAAAAGCAGGAGACCAAATTATTTGTATGGATGATGTGTATGGAGGTACAAACAGGTACTTCAGGC
AAGTGGCATCTGAATTTGGATTAAAGATTTCTTTTGTTGATTGTTCCAAAATCAAATTACTAGAGGCAGCAATTACACCAGAAACCAAGCTTGTTTGGATCGAAACCCCCA
CAAACCCCACCCAGAAGGTGATTGACATTGAAGGCTGTGCACATATTGTCCATAAGCATGGAGACATTATTTTGGTCGTGGATAACACTTTTATGTCACCATATTTCCAGC
GCCCTTTGGCTCTGGGAGCTGATATTTCTATGTATTCTGCAACAAAATACATGAATGGCCACAGTGATGTTGTAATGGGCCTGGTGTCTGTTAATTGTGAAAGCCTTCATA
ATAGACTTCGTTTCTTGCAAAACTCTCTTGGAGCAGTTCCATCTCCTATTGATTGTTACCTCTGCAATCGAGGTCTGAAGACTCTACATGTCCGAATGGAAAAGCATTTCA
AAAACGGAATGGCAGTTGCCCAGTTCCTGGAATCTAATCCTTGGGTAGAAAAGGTTATTTATCCTGGGCTGCCCTCTCATCCACAGCATGAGTTGGTGAAGCGTCAGTGTA
CAGGTTGTACAGGGATGGTCACCTTTTATATTAAGGGCACTCTTCAGCATGCTGAGATTTTCCTCAAGAACCTAAAGCTATTTACTCTGGCCGAGAGCTTGGGAGGATTCG
AAAGCCTTGCTGAGCTTCCGGCAATCATGACTCATGCATCAGTTCTTAAGAATGACAGAGATGTCCTTGGAATTAGTGACACACTGATTCGACTTTCTGTGGGCTTAGAGG
ATGAGGAAGACCTACTGGAAGATCTAGATCAAGCTTTGAAGGCAGCACACCCTCCAAGTGGATGACAGTAAAGGTGGATACGGATCCGAATAATAATAA ctcgag

>CSE_C109S
ccatgggcCATATGCACCATCATCATCATCATTCTTCTGGTGTAGATCTGGGTACCGAGAACCTGTACTTCCAATCCATGCAGGAAAAAGACGCCTCCTCACAAGGTTTCC
TGCCACACTTCCAACATTTCGCCACGCAGGCGATCCATGTGGGCCAGGATCCAGAGCAATGGACCTCCAGGGCTGTAGTGCCCCCCATCTCACTGTCCACCACGTTCAAGC
AAGGGGCGCCTGGCCAGCACTCGGGTTTTGAATATAGCCGTTCTGGAAATCCCACTAGGAATTGCCTTGAAAAAGCAGTGGCAGCACTGGATGGGGCTAAGTACTGTTTGG
CCTTTGCTTCAGGTTTAGCAGCCACTGTAACTATTACCCATCTTTTAAAAGCAGGAGACCAAATTATTAGCATGGATGATGTGTATGGAGGTACAAACAGGTACTTCAGGC
AAGTGGCATCTGAATTTGGATTAAAGATTTCTTTTGTTGATTGTTCCAAAATCAAATTACTAGAGGCAGCAATTACACCAGAAACCAAGCTTGTTTGGATCGAAACCCCCA
CAAACCCCACCCAGAAGGTGATTGACATTGAAGGCTGTGCACATATTGTCCATAAGCATGGAGACATTATTTTGGTCGTGGATAACACTTTTATGTCACCATATTTCCAGC
GCCCTTTGGCTCTGGGAGCTGATATTTCTATGTATTCTGCAACAAAATACATGAATGGCCACAGTGATGTTGTAATGGGCCTGGTGTCTGTTAATTGTGAAAGCCTTCATA
ATAGACTTCGTTTCTTGCAAAACTCTCTTGGAGCAGTTCCATCTCCTATTGATTGTTACCTCTGCAATCGAGGTCTGAAGACTCTACATGTCCGAATGGAAAAGCATTTCA
AAAACGGAATGGCAGTTGCCCAGTTCCTGGAATCTAATCCTTGGGTAGAAAAGGTTATTTATCCTGGGCTGCCCTCTCATCCACAGCATGAGTTGGTGAAGCGTCAGTGTA
CAGGTTGTACAGGGATGGTCACCTTTTATATTAAGGGCACTCTTCAGCATGCTGAGATTTTCCTCAAGAACCTAAAGCTATTTACTCTGGCCGAGAGCTTGGGAGGATTCG
AAAGCCTTGCTGAGCTTCCGGCAATCATGACTCATGCATCAGTTCTTAAGAATGACAGAGATGTCCTTGGAATTAGTGACACACTGATTCGACTTTCTGTGGGCTTAGAGG
ATGAGGAAGACCTACTGGAAGATCTAGATCAAGCTTTGAAGGCAGCACACCCTCCAAGTGGATGACAGTAAAGGTGGATACGGATCCGAATAATAATAA ctcgag

>CSE_C137S
ccatgggcCATATGCACCATCATCATCATCATTCTTCTGGTGTAGATCTGGGTACCGAGAACCTGTACTTCCAATCCATGCAGGAAAAAGACGCCTCCTCACAAGGTTTCC
TGCCACACTTCCAACATTTCGCCACGCAGGCGATCCATGTGGGCCAGGATCCAGAGCAATGGACCTCCAGGGCTGTAGTGCCCCCCATCTCACTGTCCACCACGTTCAAGC
AAGGGGCGCCTGGCCAGCACTCGGGTTTTGAATATAGCCGTTCTGGAAATCCCACTAGGAATTGCCTTGAAAAAGCAGTGGCAGCACTGGATGGGGCTAAGTACTGTTTGG
CCTTTGCTTCAGGTTTAGCAGCCACTGTAACTATTACCCATCTTTTAAAAGCAGGAGACCAAATTATTTGTATGGATGATGTGTATGGAGGTACAAACAGGTACTTCAGGC
AAGTGGCATCTGAATTTGGATTAAAGATTTCTTTTGTTGATAGCTCCAAAATCAAATTACTAGAGGCAGCAATTACACCAGAAACCAAGCTTGTTTGGATCGAAACCCCCA
CAAACCCCACCCAGAAGGTGATTGACATTGAAGGCTGTGCACATATTGTCCATAAGCATGGAGACATTATTTTGGTCGTGGATAACACTTTTATGTCACCATATTTCCAGC
GCCCTTTGGCTCTGGGAGCTGATATTTCTATGTATTCTGCAACAAAATACATGAATGGCCACAGTGATGTTGTAATGGGCCTGGTGTCTGTTAATTGTGAAAGCCTTCATA
ATAGACTTCGTTTCTTGCAAAACTCTCTTGGAGCAGTTCCATCTCCTATTGATTGTTACCTCTGCAATCGAGGTCTGAAGACTCTACATGTCCGAATGGAAAAGCATTTCA
AAAACGGAATGGCAGTTGCCCAGTTCCTGGAATCTAATCCTTGGGTAGAAAAGGTTATTTATCCTGGGCTGCCCTCTCATCCACAGCATGAGTTGGTGAAGCGTCAGTGTA
CAGGTTGTACAGGGATGGTCACCTTTTATATTAAGGGCACTCTTCAGCATGCTGAGATTTTCCTCAAGAACCTAAAGCTATTTACTCTGGCCGAGAGCTTGGGAGGATTCG
AAAGCCTTGCTGAGCTTCCGGCAATCATGACTCATGCATCAGTTCTTAAGAATGACAGAGATGTCCTTGGAATTAGTGACACACTGATTCGACTTTCTGTGGGCTTAGAGG
ATGAGGAAGACCTACTGGAAGATCTAGATCAAGCTTTGAAGGCAGCACACCCTCCAAGTGGATGACAGTAAAGGTGGATACGGATCCGAATAATAATAA ctcgag

Sequences of CSE variants inserted in pET28b between the NcoI and BamHI sites (underlined in each sequence).

>CSE_C172S
ccatgggcCATATGCACCATCATCATCATCATTCTTCTGGTGTAGATCTGGGTACCGAGAACCTGTACTTCCAATCCATGCAGGAAAAAGACGCCTCCTCACAAGGTTTCC
TGCCACACTTCCAACATTTCGCCACGCAGGCGATCCATGTGGGCCAGGATCCAGAGCAATGGACCTCCAGGGCTGTAGTGCCCCCCATCTCACTGTCCACCACGTTCAAGC
AAGGGGCGCCTGGCCAGCACTCGGGTTTTGAATATAGCCGTTCTGGAAATCCCACTAGGAATTGCCTTGAAAAAGCAGTGGCAGCACTGGATGGGGCTAAGTACTGTTTGG
CCTTTGCTTCAGGTTTAGCAGCCACTGTAACTATTACCCATCTTTTAAAAGCAGGAGACCAAATTATTTGTATGGATGATGTGTATGGAGGTACAAACAGGTACTTCAGGC
AAGTGGCATCTGAATTTGGATTAAAGATTTCTTTTGTTGATTGTTCCAAAATCAAATTACTAGAGGCAGCAATTACACCAGAAACCAAGCTTGTTTGGATCGAAACCCCCA
CAAACCCCACCCAGAAGGTGATTGACATTGAAGGCAGCGCACATATTGTCCATAAGCATGGAGACATTATTTTGGTCGTGGATAACACTTTTATGTCACCATATTTCCAGC
GCCCTTTGGCTCTGGGAGCTGATATTTCTATGTATTCTGCAACAAAATACATGAATGGCCACAGTGATGTTGTAATGGGCCTGGTGTCTGTTAATTGTGAAAGCCTTCATA
ATAGACTTCGTTTCTTGCAAAACTCTCTTGGAGCAGTTCCATCTCCTATTGATTGTTACCTCTGCAATCGAGGTCTGAAGACTCTACATGTCCGAATGGAAAAGCATTTCA
AAAACGGAATGGCAGTTGCCCAGTTCCTGGAATCTAATCCTTGGGTAGAAAAGGTTATTTATCCTGGGCTGCCCTCTCATCCACAGCATGAGTTGGTGAAGCGTCAGTGTA
CAGGTTGTACAGGGATGGTCACCTTTTATATTAAGGGCACTCTTCAGCATGCTGAGATTTTCCTCAAGAACCTAAAGCTATTTACTCTGGCCGAGAGCTTGGGAGGATTCG
AAAGCCTTGCTGAGCTTCCGGCAATCATGACTCATGCATCAGTTCTTAAGAATGACAGAGATGTCCTTGGAATTAGTGACACACTGATTCGACTTTCTGTGGGCTTAGAGG
ATGAGGAAGACCTACTGGAAGATCTAGATCAAGCTTTGAAGGCAGCACACCCTCCAAGTGGATGACAGTAAAGGTGGATACGGATCCGAATAATAATAA ctcgag

>CSE_C229S
ccatgggcCATATGCACCATCATCATCATCATTCTTCTGGTGTAGATCTGGGTACCGAGAACCTGTACTTCCAATCCATGCAGGAAAAAGACGCCTCCTCACAAGGTTTCC
TGCCACACTTCCAACATTTCGCCACGCAGGCGATCCATGTGGGCCAGGATCCAGAGCAATGGACCTCCAGGGCTGTAGTGCCCCCCATCTCACTGTCCACCACGTTCAAGC
AAGGGGCGCCTGGCCAGCACTCGGGTTTTGAATATAGCCGTTCTGGAAATCCCACTAGGAATTGCCTTGAAAAAGCAGTGGCAGCACTGGATGGGGCTAAGTACTGTTTGG
CCTTTGCTTCAGGTTTAGCAGCCACTGTAACTATTACCCATCTTTTAAAAGCAGGAGACCAAATTATTTGTATGGATGATGTGTATGGAGGTACAAACAGGTACTTCAGGC
AAGTGGCATCTGAATTTGGATTAAAGATTTCTTTTGTTGATTGTTCCAAAATCAAATTACTAGAGGCAGCAATTACACCAGAAACCAAGCTTGTTTGGATCGAAACCCCCA
CAAACCCCACCCAGAAGGTGATTGACATTGAAGGCTGTGCACATATTGTCCATAAGCATGGAGACATTATTTTGGTCGTGGATAACACTTTTATGTCACCATATTTCCAGC
GCCCTTTGGCTCTGGGAGCTGATATTTCTATGTATTCTGCAACAAAATACATGAATGGCCACAGTGATGTTGTAATGGGCCTGGTGTCTGTTAATTCTGAAAGCCTTCATA
ATAGACTTCGTTTCTTGCAAAACTCTCTTGGAGCAGTTCCATCTCCTATTGATTGTTACCTCTGCAATCGAGGTCTGAAGACTCTACATGTCCGAATGGAAAAGCATTTCA
AAAACGGAATGGCAGTTGCCCAGTTCCTGGAATCTAATCCTTGGGTAGAAAAGGTTATTTATCCTGGGCTGCCCTCTCATCCACAGCATGAGTTGGTGAAGCGTCAGTGTA
CAGGTTGTACAGGGATGGTCACCTTTTATATTAAGGGCACTCTTCAGCATGCTGAGATTTTCCTCAAGAACCTAAAGCTATTTACTCTGGCCGAGAGCTTGGGAGGATTCG
AAAGCCTTGCTGAGCTTCCGGCAATCATGACTCATGCATCAGTTCTTAAGAATGACAGAGATGTCCTTGGAATTAGTGACACACTGATTCGACTTTCTGTGGGCTTAGAGG
ATGAGGAAGACCTACTGGAAGATCTAGATCAAGCTTTGAAGGCAGCACACCCTCCAAGTGGATGACAGTAAAGGTGGATACGGATCCGAATAATAATAA ctcgag

>CSE_C307S
ccatgggcCATATGCACCATCATCATCATCATTCTTCTGGTGTAGATCTGGGTACCGAGAACCTGTACTTCCAATCCATGCAGGAAAAAGACGCCTCCTCACAAGGTTTCC
TGCCACACTTCCAACATTTCGCCACGCAGGCGATCCATGTGGGCCAGGATCCAGAGCAATGGACCTCCAGGGCTGTAGTGCCCCCCATCTCACTGTCCACCACGTTCAAGC
AAGGGGCGCCTGGCCAGCACTCGGGTTTTGAATATAGCCGTTCTGGAAATCCCACTAGGAATTGCCTTGAAAAAGCAGTGGCAGCACTGGATGGGGCTAAGTACTGTTTGG
CCTTTGCTTCAGGTTTAGCAGCCACTGTAACTATTACCCATCTTTTAAAAGCAGGAGACCAAATTATTTGTATGGATGATGTGTATGGAGGTACAAACAGGTACTTCAGGC
AAGTGGCATCTGAATTTGGATTAAAGATTTCTTTTGTTGATTGTTCCAAAATCAAATTACTAGAGGCAGCAATTACACCAGAAACCAAGCTTGTTTGGATCGAAACCCCCA
CAAACCCCACCCAGAAGGTGATTGACATTGAAGGCTGTGCACATATTGTCCATAAGCATGGAGACATTATTTTGGTCGTGGATAACACTTTTATGTCACCATATTTCCAGC
GCCCTTTGGCTCTGGGAGCTGATATTTCTATGTATTCTGCAACAAAATACATGAATGGCCACAGTGATGTTGTAATGGGCCTGGTGTCTGTTAATTGTGAAAGCCTTCATA
ATAGACTTCGTTTCTTGCAAAACTCTCTTGGAGCAGTTCCATCTCCTATTGATTGTTACCTCTGCAATCGAGGTCTGAAGACTCTACATGTCCGAATGGAAAAGCATTTCA
AAAACGGAATGGCAGTTGCCCAGTTCCTGGAATCTAATCCTTGGGTAGAAAAGGTTATTTATCCTGGGCTGCCCTCTCATCCACAGCATGAGTTGGTGAAGCGTCAGAGCA
CAGGTTGTACAGGGATGGTCACCTTTTATATTAAGGGCACTCTTCAGCATGCTGAGATTTTCCTCAAGAACCTAAAGCTATTTACTCTGGCCGAGAGCTTGGGAGGATTCG
AAAGCCTTGCTGAGCTTCCGGCAATCATGACTCATGCATCAGTTCTTAAGAATGACAGAGATGTCCTTGGAATTAGTGACACACTGATTCGACTTTCTGTGGGCTTAGAGG
ATGAGGAAGACCTACTGGAAGATCTAGATCAAGCTTTGAAGGCAGCACACCCTCCAAGTGGATGACAGTAAAGGTGGATACGGATCCGAATAATAATAA ctcgag

>CSE_C310S
ccatgggcCATATGCACCATCATCATCATCATTCTTCTGGTGTAGATCTGGGTACCGAGAACCTGTACTTCCAATCCATGCAGGAAAAAGACGCCTCCTCACAAGGTTTCC
TGCCACACTTCCAACATTTCGCCACGCAGGCGATCCATGTGGGCCAGGATCCAGAGCAATGGACCTCCAGGGCTGTAGTGCCCCCCATCTCACTGTCCACCACGTTCAAGC
AAGGGGCGCCTGGCCAGCACTCGGGTTTTGAATATAGCCGTTCTGGAAATCCCACTAGGAATTGCCTTGAAAAAGCAGTGGCAGCACTGGATGGGGCTAAGTACTGTTTGG
CCTTTGCTTCAGGTTTAGCAGCCACTGTAACTATTACCCATCTTTTAAAAGCAGGAGACCAAATTATTTGTATGGATGATGTGTATGGAGGTACAAACAGGTACTTCAGGC
AAGTGGCATCTGAATTTGGATTAAAGATTTCTTTTGTTGATTGTTCCAAAATCAAATTACTAGAGGCAGCAATTACACCAGAAACCAAGCTTGTTTGGATCGAAACCCCCA
CAAACCCCACCCAGAAGGTGATTGACATTGAAGGCTGTGCACATATTGTCCATAAGCATGGAGACATTATTTTGGTCGTGGATAACACTTTTATGTCACCATATTTCCAGC
GCCCTTTGGCTCTGGGAGCTGATATTTCTATGTATTCTGCAACAAAATACATGAATGGCCACAGTGATGTTGTAATGGGCCTGGTGTCTGTTAATTGTGAAAGCCTTCATA
ATAGACTTCGTTTCTTGCAAAACTCTCTTGGAGCAGTTCCATCTCCTATTGATTGTTACCTCTGCAATCGAGGTCTGAAGACTCTACATGTCCGAATGGAAAAGCATTTCA
AAAACGGAATGGCAGTTGCCCAGTTCCTGGAATCTAATCCTTGGGTAGAAAAGGTTATTTATCCTGGGCTGCCCTCTCATCCACAGCATGAGTTGGTGAAGCGTCAGTGTA
CAGGTAGCACAGGGATGGTCACCTTTTATATTAAGGGCACTCTTCAGCATGCTGAGATTTTCCTCAAGAACCTAAAGCTATTTACTCTGGCCGAGAGCTTGGGAGGATTCG
AAAGCCTTGCTGAGCTTCCGGCAATCATGACTCATGCATCAGTTCTTAAGAATGACAGAGATGTCCTTGGAATTAGTGACACACTGATTCGACTTTCTGTGGGCTTAGAGG
ATGAGGAAGACCTACTGGAAGATCTAGATCAAGCTTTGAAGGCAGCACACCCTCCAAGTGGATGACAGTAAAGGTGGATACGGATCCGAATAATAATAA ctcgag
